# Behavioral Theories That Have Influenced the Way Health State Preferences Are Elicited and Interpreted: A Bibliometric Mapping Analysis of the Time Trade-Off Method With VOSviewer Visualization

**DOI:** 10.3389/frhs.2022.848087

**Published:** 2022-07-01

**Authors:** Luke Henstock, Ruth Wong, Aki Tsuchiya, Anne Spencer

**Affiliations:** ^1^University of Birmingham, Birmingham, United Kingdom; ^2^School of Health and Related Research, University of Sheffield, Sheffield, United Kingdom; ^3^Department of Economics and School of Health and Related Research, University of Sheffield, Sheffield, United Kingdom; ^4^Department of Health and Community Sciences, University of Exeter, Exeter, United Kingdom

**Keywords:** Prospect Theory, time trade off, evidence synthesis, Non-Expected Utility Theory, Expected Utility Theory, Probabilistic Choice Theory, Order Effects, visualization

## Abstract

**Aim:**

The aim of this paper is to develop an understanding of how behavioral theories have influenced the way preferences for health-related quality of life are elicited and interpreted. We focus on the Time Trade-off (TTO) method given it represents the quality-adjusted life-year (QALY) concept—that survival in less-than-full health can be deemed equivalent to a shorter survival in full health. To our knowledge this is the first review using a combination of systematic scoping review, bibliometrics and VOSviewer visualization to map the development of ideas in health economics.

**Methods:**

A priori, we selected three behavioral theories to explore within our review, referred to here as Expected Utility Theory, Non-Expected Utility Theory and Probabilistic Choice Theory. A fourth topic, Order Effects, is defined broadly to encompass behavioral theories around timing/sequence of events. For the main search, Scopus was used to identify literature that had (a) elicited TTO values and/or (b) contributed to the way TTO values were elicited and interpreted, from inception to July 2021. Papers that focused on the latter category were given the label “behavioral” and underwent additional analyses. A two stage-screening was applied to assess eligibility. Co-citation, co-authorship and co-occurrence of keywords was used to chart the development of TTO over time.

**Results:**

A total of 1,727 records were retrieved from Scopus and were supplemented by an additional 188 papers. There were 856 applied and 280 behavioral papers included in the final corpus, with the behavioral set split equally into four sets of 70 papers to chart the development of keywords over time: (1) 1972–1999; (2) 2000–2010, (3) 2010–2015 and (4) 2015–2021.

**Discussion:**

The keyword analysis suggested that whilst some ideas transition quickly from economic theory to the TTO literature, such as the impact of Order Effects, others take longer to be assimilated, for example Non-Expected Utility models or failure of constant discounting. It is therefore important that researchers within health economics work more closely with those in mainstream economics and keep abreast of the wider economics and behavioral sciences to expedite the uptake of new and relevant ideas.

## Introduction

The quality-adjusted life year (QALY) amalgamates life expectancy with a preference-based score of health-related quality of life, providing a single, internationally recognized index measure of health (1). The QALY has also come to form a central part of many countries' Health Technology Appraisal systems, such as the UK, Netherlands, Canada, Australia, and New Zealand (2). In the US, the QALY has transcended academia and is commonly adopted by non-profit organizations, but it currently has little influence on public health policy (3).

Our understanding of what the QALY represents, and the elicitation methods used to reveal individual preferences has been subject to many changes since the concept was first developed. To explore these changes, we focus on the Time Trade-off (TTO) method given it represents the QALY concept—that survival in less than full health can be deemed equivalent to a shorter survival in full health (4–6). The TTO method for health has been attributed to Fanshel and Bush (7), and Torrance et al. (8) and has long been the most widely used method for eliciting preferences (4, 9). However, the methods used to elicit TTO values vary (10, 11).

Our understanding of the behavioral theories underpinning preference elicitation has changed over time. Pliskin et al. (12) defined the axioms for the TTO and Standard Gamble (SG) to be considered as a measure of von Neumann and Morgenstern expected utility functions. These axioms were: (1) utility independence between quality and length of life; (2) constant proportional trade of length and quality of life and (3) risk neutrality of life years implying a linear utility of life duration. Prospect Theory (13) introduced the notion of a reference point and losses and gains relative to this reference point. In this theory, loss aversion assigns larger weight to losses compared to gains. Prospect Theory also introduced the notion of overweighting small probabilities and underweighting of large probabilities. Loewenstein and Prelec (14) and Chapman et al. (15) highlighted that people may have preferences over the order or sequence in which events occur over time, leading to papers looking at the limitations imposed by assuming you can simply add the utilities from constituent health states when health varies over time (16, 17). Elicitation procedures may also unintentionally introduce Order Effects, such as the order in which the states were valued (10, 18). Finally, an additional innovation, inspired by behavioral theories was Probabilistic Choice, termed Random Utility Theory (19), which superseded deterministic choice. Random Utility Theory underpins Discrete Choice Experiments (DCEs) (20) to elicit and analyze ordinal data to elicit preferences.

Previous comprehensive literature reviews have covered important aspects of the QALY methodology (1, 21–24). There are also reviews of particular applications, such as DCEs to estimate health state values (25), as well as meta-analyses of the impacts of study characteristics and elicitation methods on values (26) and biases arising in health state measurement (27). There are further reviews and empirical estimations of the thresholds for cost-effectiveness analysis (28) and reviews that explore the interplay between politics, policy, and the challenges operationalizing the QALY concept in countries such as the UK (29). However, to date, we are aware of just one paper by Spencer et al. (30) that covered the uptake of behavioral theories that have influenced the way health state preferences are elicited. Spencer et al. (30) used bibliometric and visualization techniques to identify and explore the uptake of Fanshel and Bush (7), and Torrance et al. (8) over time within the health state valuation literature. Bibliometrics and visualization techniques are forming new methods to review large scientific databases, enabling researchers to create scientific maps reflecting the links between authors, references, and keywords to gain an overall picture of the evidence base (31, 32).

Spencer et al.'s review focused on non-clinical papers to outline the range of themes discussed, with early papers assessing how to measure the quality of care in hospitals and exploring classification systems to describe health states to later papers discussing considerations of equity. They further went on to explore the proliferation of theories drawn from decision science and economics—Expected Utility Theory (33), Non-Expected Utility Theory (13, 14, 34) and Probabilistic Choice Theory (19) and a fourth category, Order Effects, covering a range of behavioral theories around timing/sequence of events (14). However, a weakness of Spencer's review was that only papers that cite the source papers were included. Moreover, the review acknowledged that different authors do not necessarily base referencing decisions on a shared understanding of the relevant literature, so the link between the sets of references cited and the research questions addressed was not necessarily strong. Consequently, they were unable to map the development of themes over time using visualizations, as the themes could not easily be traced over time.

The aim of this paper is to extend Spencer et al. (30) review by using a novel approach that combines systematic scoping review with bibliometric and visualization techniques, referred to as research weaving, to facilitate a high-level approach (35) combined with a broader search strategy to capture the wider corpus of the literature relating to TTO. Within this review we aim to explore how behavioral theories have influenced the way preferences for health-related quality of life are elicited and interpreted using bibliometric co-citation at an author, paper and geographic level. Furthermore, to allow a more granular interpretation of the papers, and in particular, the development of behavioral topics over time, we conduct analysis of author and reviewer-added keywords to explore how these keys words have changed over time.

## Methods

This study followed the Preferred Reporting Items for Systematic reviews and Meta-Analyses extension for Scoping Reviews (PRISMA-ScR) updated guidelines developed by Tricco et al. (36).

The corpus of papers was formed in two stages: stage one involved the use of a broad search strategy to capture the wider corpus of the literature relating to TTO and stage two supplemented the search with citation searches. In creating a map, VOSviewer can support several file types and handle multiple files from a single database such as PubMed, Web of Science, and Scopus. However, the map can only be derived from a single source and it is not possible to merge filetypes from several sources to generate a map. We chose Scopus, a multidisciplinary database that also includes records from a range of sources including MEDLINE and Embase.

An electronic database search of Scopus was carried out in order to identify articles containing TTO in the title and/or abstract: (TITLE ((“time trade off” OR “time trade-off” OR “time tradeoff”)) OR ABS ((“time trade off” OR “time trade-off” OR “time tradeoff”))) AND (LIMIT-TO (DOCTYPE ,“ar”)) from database inception to 20th June 2021. The search strategy was kept relatively broad to maximize the likelihood of identifying all relevant publications. The search was not limited by language, but it was limited to journal articles, denoted by “ar” in the search, thus excluding book chapters, conference abstracts, and gray literature. EndNote (version 9.3.3) was used to check for and remove duplicates (37). Given the cross-disciplinary nature of the search, we did not utilize subject areas option in Scopus or use semi-automated searches to limit the scope of the search as others have done (38, 39), and instead chose to manually apply inclusion and exclusion criteria.

In stage two, we included the set of papers drawn from the non-clinical journals that cited either Torrance et al. (8) or Fanshel and Bush (7), or both. These papers were identified in Spencer et al. (30) based on a search of the Web of Science Core Collection (Clarivate Analytics) from inception to October 2020. Stage two, therefore, identified additional papers that elicited TTO values but that did not include “time trade-off” or other derivatives of this term within the title or abstract. Given the success of the Spencer search, Fanshel and Bush (7) and Torrance et al. (8) were deemed sufficient as source papers for the purposes of our search.

## Screening and Eligibility

At the first stage, journal and titles were screened in EndNote manually (AS, LH and RW) for inclusion: papers that have used the term “time trade-off” that were not relevant to health care, or health state utilities, were excluded. It was anticipated that this step would lead to the largest number of exclusions, due to the use of the term ‘time trade-off' for optimizing mathematical models in other scientific domains and disciplines, such as computer science and engineering (40). We then added a subset of the papers published in the non-clinical journals that were reviewed and included in a cluster analysis by Spencer et al. (30).

The first stage allowed us to explore the types of papers that were falling within the search, to help develop and refine the inclusion and exclusion criteria for use in the second stage. This criterion was formed iteratively, using a subset of papers initially to identify and label broad topics, followed by a discussion with all authors on which topics to retain to hone down on literature to focus on the implementation of the TTO and methodological papers. The final inclusion criteria included papers that elicited TTO data and performed a secondary analysis on TTO data, or outlined conceptual factors affecting the TTO methods, as shown in Table 1.

**Table 1:** Inclusion and exclusion criteria for papers.

**Inclusion criteria**	**Exclusion criteria**
– Where respondents have completed a TTO questionnaire.	– Apply the TTO tariff to self-reported EQ-5D classification collected within the study, but do not elicit the TTO values (denoted by ‘applied TTO tariff' in the keywords).
– Where authors have performed secondary data analysis on raw TTO values to explore, for example, the econometric model that is applied to TTO values [example: Craig (41), e.g., Stolk secondary analysis of how TTO methods impact on values].	– Conduct economic evaluations or decision analytic models including utility values, but do not elicit values using a TTO question as part of the study, or do not report in sufficient detail the TTO question used (identified with the label ‘economic evaluation' and/or ‘decision analytic model).
– Theoretical papers and review papers that outline the conceptual factors affecting the TTO methods.	– Conduct secondary data analysis of TTO data sets—for example international comparisons of TTO values using pooled data, and/or creating normative benchmark values weighted by population from pooled data (42) but do not explore the extent to which different TTO methods can explain the difference in valuations (identified with the label ‘international' and/or ‘international comparison').
	– Reviews or systematic reviews of TTO values in a clinical area—that do not aim to explore the behavioral/methodological aspects of the methods (identified with the label ‘systematic review').
	– Online study protocols.

Two reviewers (AS and LH) independently screened papers using these inclusion and exclusion criteria. An additional set of inclusion criteria were used to differentiate between papers that drawn on behavioral theories (Table 2), and others that were applications of such theories. This approach identified a “behavioral” set and an “applied” set of papers for further analysis (43).

**Table 2:** Inclusion criteria for the set of methodological paper.

**Articles are deemed to be behavioral if:**
– Test the axioms of expected utility, and, or Non-Expected Utility
– Test procedural invariance (e.g., change method of elicitation and should not change values), including variation in procedures to iterate toward value
– Explore the impacts of sequence and ordering effects of health states
– Explore the impact of random utility and choice to operationalise the TTO [e.g., DCE (TTO)]

## Bibliometric Analysis and Visualizations

Co-citation analysis measures the relationship between authors, papers, organizations and countries based on the number of documents they (authors, papers, organizations or countries) co-authored together. Similarly, a co-occurrence analysis measures the relationship between keywords, or “scientific terms,” that occur together in the same paper (44).

The visualizations were created in VOSviewer from bibliometric outputs generated from either Scopus or Endnote. In a VOSviewer map that is used to visualize these relationships, the size of a given node (keyword or author or specific paper or country) depends on their relative importance. For example, the position and size of a keyword in a co-occurrence analysis depends on its relative centrality and strength, determined by the number of times it occurs in the literature and how many times it co-occurs with other keywords. The keywords with the greatest weight are represented as the largest nodes in a network analysis (45). Within co-citation analyses (looking at authors and specific papers), centrality within a map indicates the central power of a node, and how important an author or article is to the rest of the network. Similarly, the strength of a node is indicated by the thickness of a line between two nodes and varies depending on the number of articles that co-cites the two nodes (46).

The myriad functionalities and flexibility of VOSviewer allow us to create network (number of connections by clusters), overlay (temporal analysis) and density visualizations (uses a color scheme to denote occurrences rather than clusters). This deepens our understanding of the relationships in the literature compared to using one form of mapping in isolation.

There is some judgement needed on setting the threshold for the minimum number of citations for visualization. A low threshold allows more items to be analyzed and displayed on the map, but also may create overly complex images. Whilst VOSviewer applies techniques to optimize the visualization so that labels of nodes do not overlap each other, for the analysis and interpretation, it was necessary to apply a threshold to limit the number of nodes displayed in the map for clarity. Applying thresholds is a means to control the VOSviewer's viewing capabilities e.g., adjust the number of clusters and nodes displayed to give a clear visualization of the individual nodes and networks. Consequently, the threshold for the minimum number of citations was not determined a priori, as these need to be tailored to the size of the literature identified and included in the analysis. All visualizations exploring keywords over-time used the same thresholds, to allow comparisons to be drawn.

Bibliometric data from Scopus allows us to use the full array of tools available in VOSviewer for analyzing the publications. Co-authorship analysis can also be extended to explore other units of analysis, such as geographical and organizational (based on the number of publications). In analyzing the number of publications per country, we were able to establish whether the same countries that are using TTO and the QALY in its cost effectiveness strategies, are the ones contributing to its development.

For co-occurrence and co-authorship analyses, the full counting approach was used, where a publication with 6 authors leads to the creation of 5 distinct co-authorship links for each author, each with a weight of 1. The alternative approach, fractional counting, assigns a weight of 1/5th to each of the links. However, the full counting method remains the conventional approach (47).

## Keyword Analysis

Keywords analysis can represent one of the most effective ways of understanding the developments in literature over time (48). Every time two subject terms appear together in a publication's keywords they are linked together.

At an early stage it was realized that a challenge to operationalizing keyword analysis in this application was that keywords were missing from some of the earlier papers. For example, in Torrance's first two available works on Scopus both include indexed EMTREE medical terms, but do not include author keywords (8, 49). In contrast, his most recent articles include 3 (2020) and 12 (2019) keywords respectively. Therefore, to reduce bias toward newer articles that include relatively more keywords, two reviewers (AS and LH) assigned reviewer-added keywords to each article in EndNote based on a predefined keyword categorization framework (Appendix 1 in Supplementary Material).

Reviewer-added keywords were added to capture the content of the article based on the title and abstract and ranged from how the methods have been used, to specific methods, such as Healthy Years-Equivalent. The reviewer-added keywords additionally aimed to capture the theories drawn from decision science and economics, including the specific aspects considered e.g., tests of particular axioms. Using such a broad approach to reviewer-added keywords enabled us to capture not only explicit mention of behavioral theories, but also methods that had been developed in response to these theories (e.g., DCEtto, Lead Time TTO and composite TTO). Within the categorization, themes, sub-themes and reclassified keywords were used to increase precision and make sure the future co-occurrence analysis was as accurate as possible. The framework underwent several iterations and revisions during the planning stage of this study and was agreed upon after discussion between all authors. To limit selection bias, reviewers were blind to the author keywords, but reviewed them after selection to make sure there were no duplicates.

In our analysis we ensured a distinction between author, indexed and reviewer-added keywords. According to Scopus, author keywords are chosen by the author(s) reflecting the author's voice, whereas indexed keywords are chosen by content suppliers based on “publicly available vocabularies” (50). Scopus includes both author and indexed keywords and there is no upper limit for the number of keywords per paper. Typically, there are more indexed keywords than author keywords.

Keywords were merged and standardized in EndNote, to stop multiple words or phrases appearing in the visualizations that carry the same meaning. For example, “time trade off,” “time trade-off,” “time-trade-off” and “TTO” were merged under a single primary term, “TTO,” thus they only counted once (51). The steps taken to merge, standardize and de-duplicate the keywords meant our data were sufficiently clean to be used in a network analysis.

The two sets of final publications from the first and second search were combined into a single EndNote library, where the full set of author keywords were retrieved from Scopus. To explore the incorporation of behavioral theories, we investigated the development of keywords over time within the behavioral set. In this section, we split the literature into four sections, with equal numbers of papers within each section and equal citation thresholds, to explore the development of keywords over time.

## Results

### Search Results

**Figure 1:**
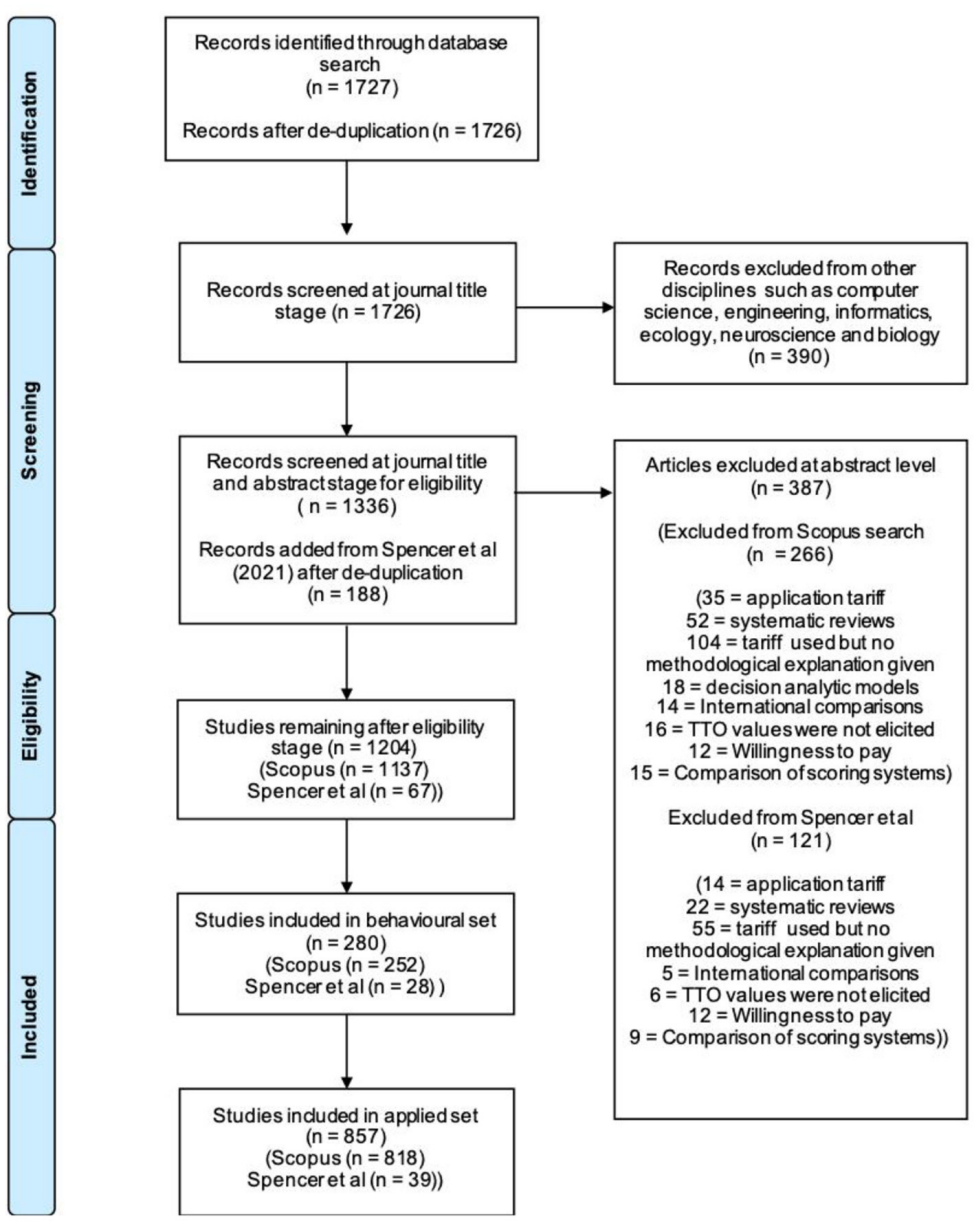
PRISMA flowchart describing the study selection process.

The online database search of Scopus retrieved a total of 1,727 records (Figure 1). Following de-duplication in EndNote, 1,726 records remained. Screening at the journal-title level led to the exclusion of 390 records as they were not related to health care or health economics. Thus, 1,336 articles remained from the database search. We then added the 204 papers across non-clinical journals that were identified and reviewed by Spencer et al. (30). A total of 16 articles were present in both sets, and after de-duplication 188 articles remained from Spencer et al. (30). Following the screening of the article title and abstract, a further 387 records were excluded due to not satisfying at least one of the inclusion criteria (261 excluded from the Scopus search and 116 excluded from Spencer et al. and 5 of the overlapping papers) (Table 1). The most common reason for the exclusion of a paper was that they used an existing TTO population value set (a specialist catalog of health state values), but a methodological explanation or justification of the methods used was not given or ambiguous. After the two screening stages, articles from the Scopus search and the 2021 search were treated as equal. After the predefined criteria (Table 1) were applied, 280 articles were identified as behavioral (criteria detailed in Table 2), and the remaining 857 articles were applied papers.

**Figure 2:**
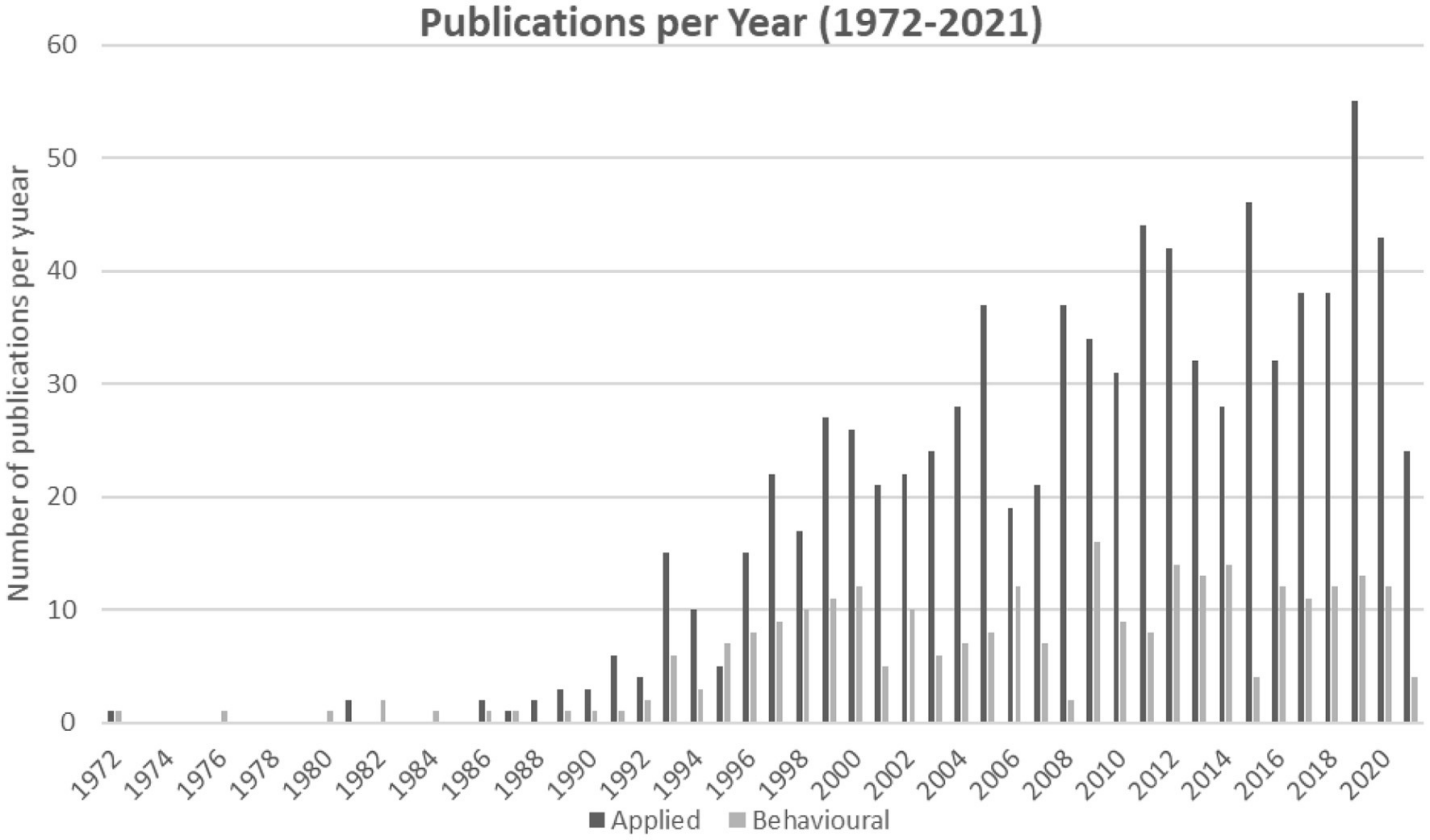
Publication trend, publications per year for the behavioral and applied sets.

The number of behavioral articles published in an individual year peaked in 2009 (*n* = 16). By comparison, the number of applied articles published in a year reached its height in 2019 (*n* = 55) and had been increasing year-on-year since 2016 (see Figure 2). The “*Medical Decision Making”* journal had the highest number of publications in the behavioral set (*n* = 48), representing 17.14% of the total behavioral set. Similarly, Medical Decision Making also had the highest number of publications in the applied set (*n* = 52), although this only represented 6.07% of the total applied set. A total of 643 authors were present in the behavioral set. Arthur E. Attema had the greatest number of articles, with 16 of his 20 total papers published in “*Medical Decision Making”* (*n* = 16). These 16 papers were all identified from the Scopus search. A total of 3,190 authors were present in the applied set of articles. The authors with the greatest number of articles were Gary C. Brown (*n* = 34) and Melissa M. Brown (*n* = 34), whose research is often collaborative and often occurs together, but neither author had an article in the behavioral set.

### Bibliometric Analysis and Visualizations

#### Country-Level Analysis

There was a total of 31 countries (or territories, used interchangeably) within the behavioral set, and 3 papers remained “unidentified” in Scopus, meaning a country could not be retrieved. The countries with the greatest number of records in the behavioral set were the Netherlands and the United States (both, *n* = 85), followed by the United Kingdom (*n* = 81). A paper can be attributed to more than one country and thus a paper could be attributed to both the Netherlands and the United States. These relationships are explored in the co-authorship of countries analysis, to assess the nature of international collaboration. The full list of papers included in the behavioral set is in Appendix 4 in Supplementary Material.

**Figure 3:**
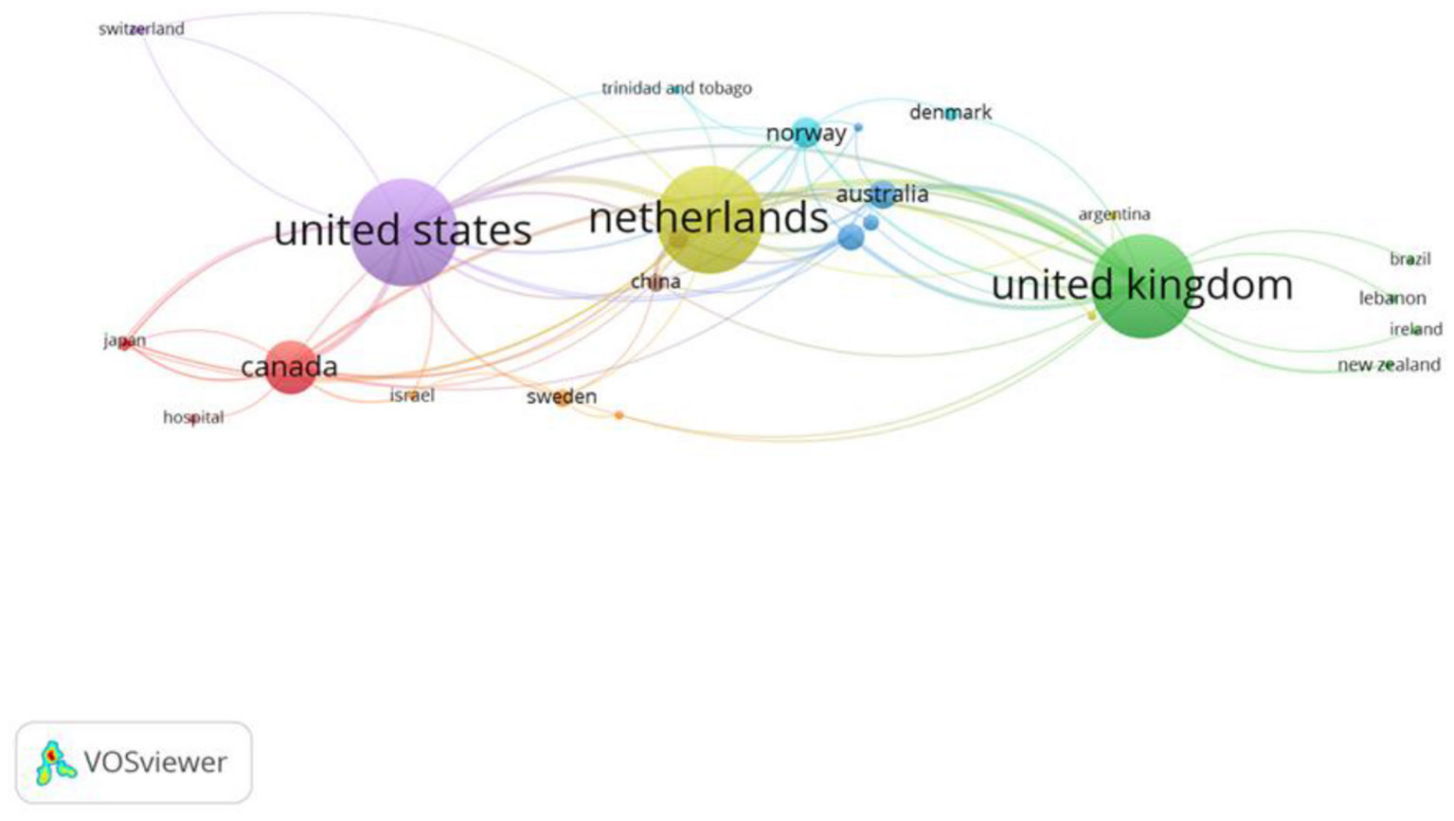
Network visualization of the co-authorship between countries, in the behavioral set, threshold = 1.

Figure 3 reports co-authorship at a country level. The Netherlands, the United States, and the United Kingdom were previously identified as the countries with the greatest number of publications, and the co-authorship analysis shows they are also the best-connected based on the total link strength. However, despite having the most articles and greatest total link strength, the Netherlands only had the fourth most citations (2,914 citations). By comparison, Canada (6,571 citations) contributed 33 articles to the behavioral set (52 fewer than the Netherlands), yet it had the second highest number of citations (behind the United Kingdom, 6,855 citations). Canada had the highest number of citations per document, on average each paper in the set was cited 199 times. The only other country that had an average number of citations per paper above 100 was Singapore, with 124 citations.

#### Author-Level Analysis

**Figure 4:**
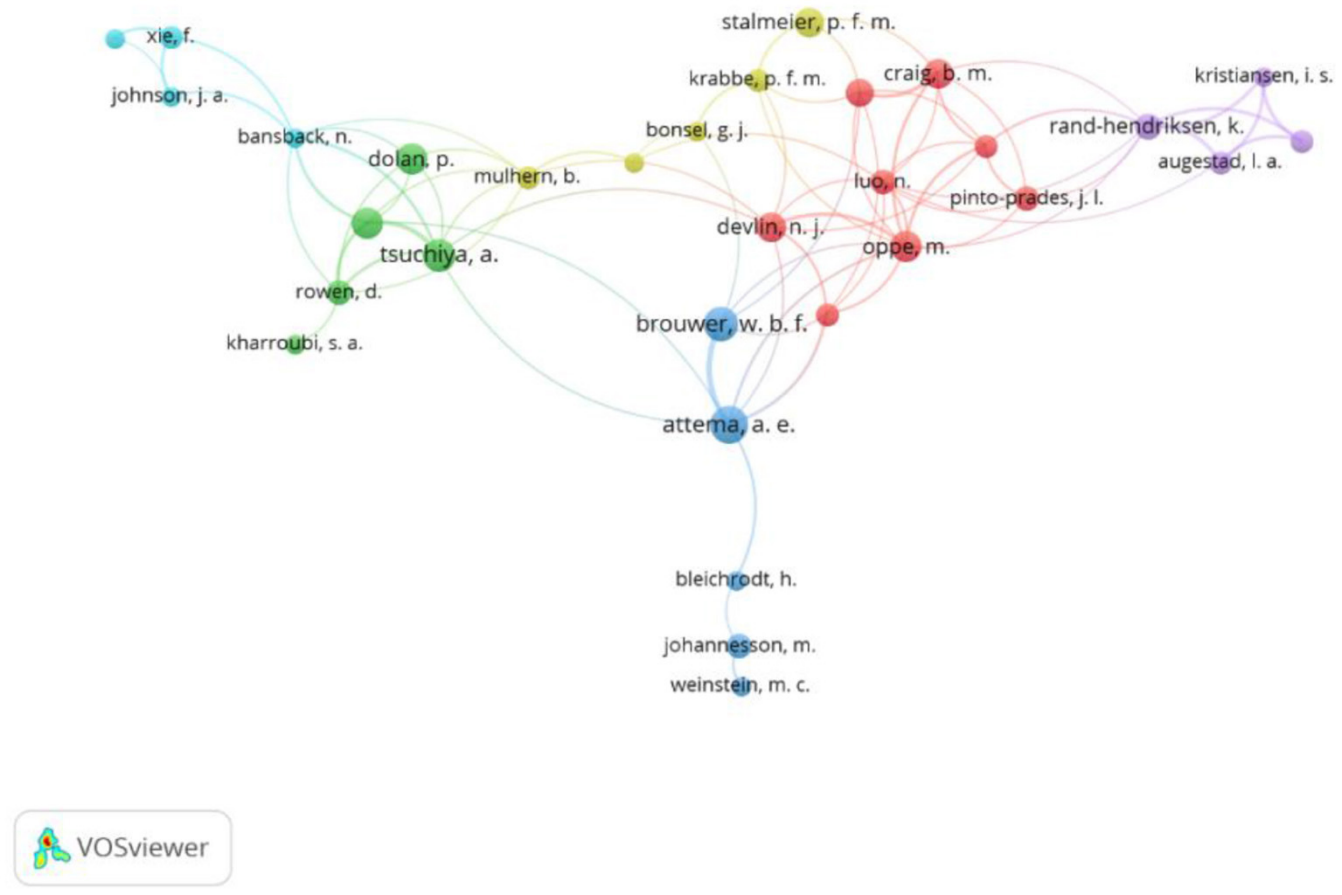
Network visualization of the co-authorship of authors, in the behavioral set, threshold = 5.

There were 643 authors present in the behavioral set. To be included in the visualizations, authors had to have five or more papers; 37 authors met this inclusion threshold. Of these 37 authors, 31 were connected and are represented in Figure 4. In this analysis, the size of the node reflects the number of documents. Attema (16) and Brouwer (14) had the most documents in the behavioral set, working on 11 papers together. Their earliest paper in 2009 described a new method for correcting TTO scores, by considering scores for utility of life duration curvature.

#### Paper-Level Analysis

**Figure 5:**
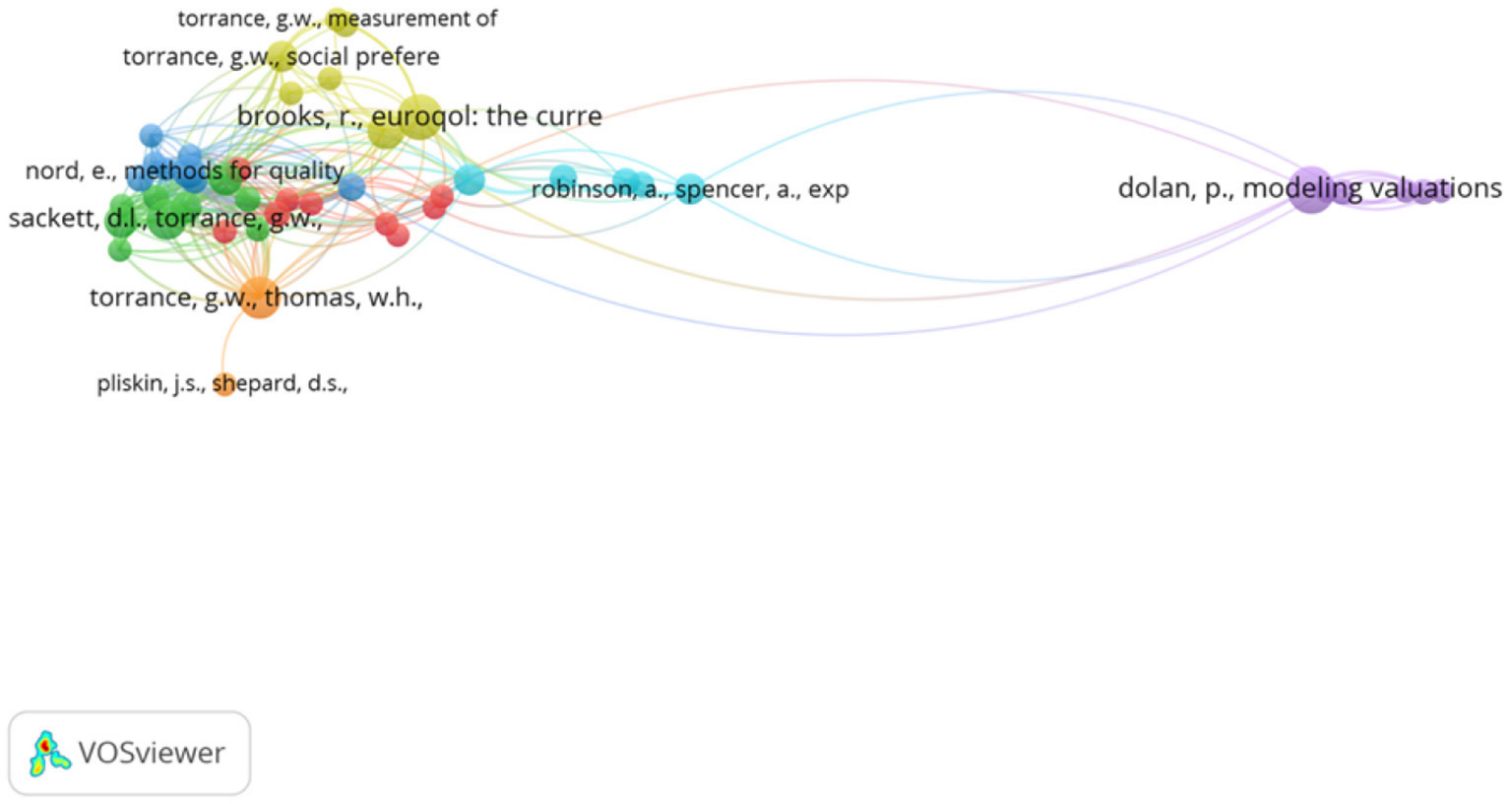
Network visualization map for the co-citation of references, in the behavioral set, threshold = 5.

Analyzing the behavioral set at the paper level allows the significance of specific papers to be understood. Figure 5 shows the network visualization map for the co-citation of references, where each node represents a publication and a link between two nodes means they were cited together by a third paper (52). For a paper to be included it must be cited a minimum of 5 times within the 280 methodological papers. Between the 280 papers, there were 8,034 cited references and 49 met the threshold, of which 46 were connected and shown in the visualization. Among the 46 publications shown, “Modeling valuations for EuroQol health states” by Dolan (53) was the most highly co-cited article (20 co-citations) and its location in relative isolation on the right of the map, whilst still being highly cited illustrates its importance and how it has been referenced in the literature as a key, stand-alone component of TTO.

**Figure 6:**
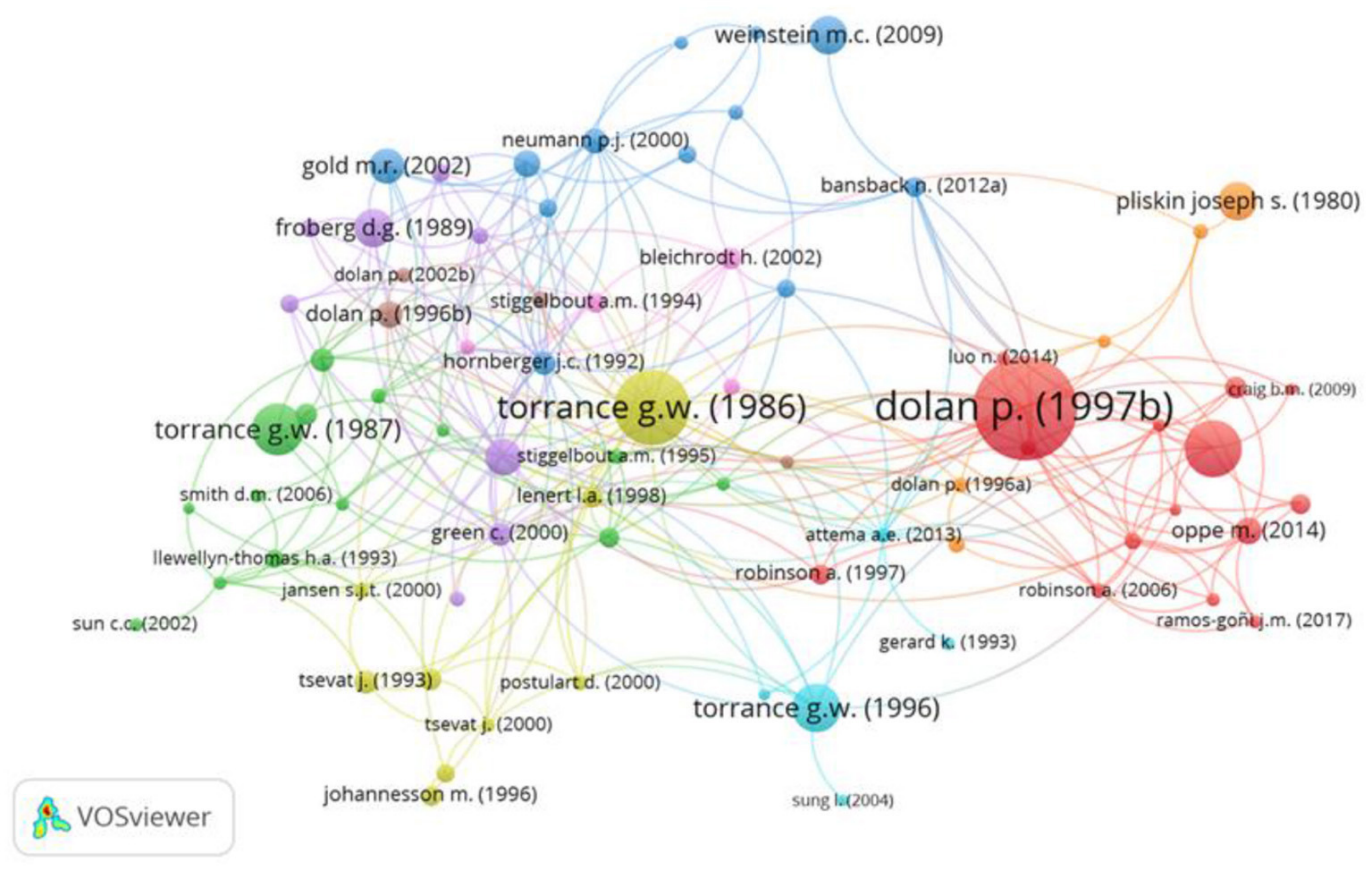
Network visualization map for the citation of documents, in the behavioral set, threshold = 50.

Further, the importance of Dolan's 1997 paper (1997 in the analysis) was supported through a citation of documents analysis. Citation analysis assesses the number of links from one article to another, but this is not limited to citations from within the behavioral set. The analysis utilizes our wider set including the 857 applied references and a minimum threshold of 50 citations was used. Figure 6 shows the citation analysis, 84 papers satisfied the threshold, but only 76 were connected. Dolan (53) is one of the most important articles within the development of the TTO methodology the topic, but various works by Torrance were also highlighted.

#### Keywords Development and Classification

Reviewer-added keywords provide additional information where a paper did not have keywords, or where the existing keywords did not fully summarize title or abstract content. Reviewer-added keywords were also necessary in cases where author keywords were not used consistently across different authors and therefore could have multiple meanings. For example, the keyword “methodological” covered a broad range of topics, from the development of classification systems and/or national tariffs.

Keywords were added to capture the content of the article based on the title and abstract. Keywords that captured the content of the article included: (1) how the methods have been used (e.g., for risk-benefit trade-offs, cost-benefit analysis, calculation of QALYs, cost-effectiveness), (2) if the study informed the development a tariff, or was exploring measurement properties (psychometrics, qualitative), (3) the domains covered (process or outcome or experience utility etc.) or who were asked (patients, member of general population), and (4) what they were asked (e.g., value own health state, value observed patient state, value experienced treatment delivery). Keywords additionally included the preference method such as TTO or SG, or if the study used more specific methods, such as Healthy Years-Equivalents and the annual profile method that value multi-state health profiles and can accommodate preferences for the order of health states. Methods such as Lead Time TTO or composite TTO that use a Lead Time to value states worse than dead aim to standardize order in which the state worse than dead and the state of full health are placed within the health profile being elicited.

The reviewer-added keywords additionally captured the theories drawn from decision science and economics, including the specific aspects considered (e.g., tests of particular axioms) such as: Expected Utility Theory (31): tests of the TTO axioms under Expected Utility Theory [(e.g., constant proportional trade-off, maximal endurable time, transitivity etc, discounting) (e.g. stationarity, increasing impatience, utility of life, aggregation across individuals) (e.g., interpersonal comparisons, or domains) (e.g. additive independence of health states and utility independence)].

Non-Expected Utility Theory (13, 14, 34) included: (1) biases, heuristics, (2) procedural invariance, within elicitation procedure and varying the elicitation procedure, (3) framing, (4) Prospect Theory, and (5) imprecision. Articles discussing adjusting values to take account of Non-Expected Utility Theory were given the keyword corrective procedures. Probabilistic Choice Theory (19) included DCEs, scalability of ranked methods, conjoint analysis, anchoring the values on the full health-dead scale and best worse scaling. Order Effects, covering a range of behavioral theories around timing/sequence of events (14) included order of states and states worse than dead.

### Keyword Analysis Over Time

We explored the development of keywords over time within the behavioral set. For this, we split the papers into equally into four sets of 70 papers each, to chart the development of keywords over time: (1) 1972–1999; (2) 2000–2010, (3) 2010–2015 and (4) 2015–2021. Throughout this analysis VOSviewer performed a cluster analysis to identify clusters of closely related keywords (54), with related clusters shown in different colors.

#### Keywords: Period 1972–1999

**Figure 7:**
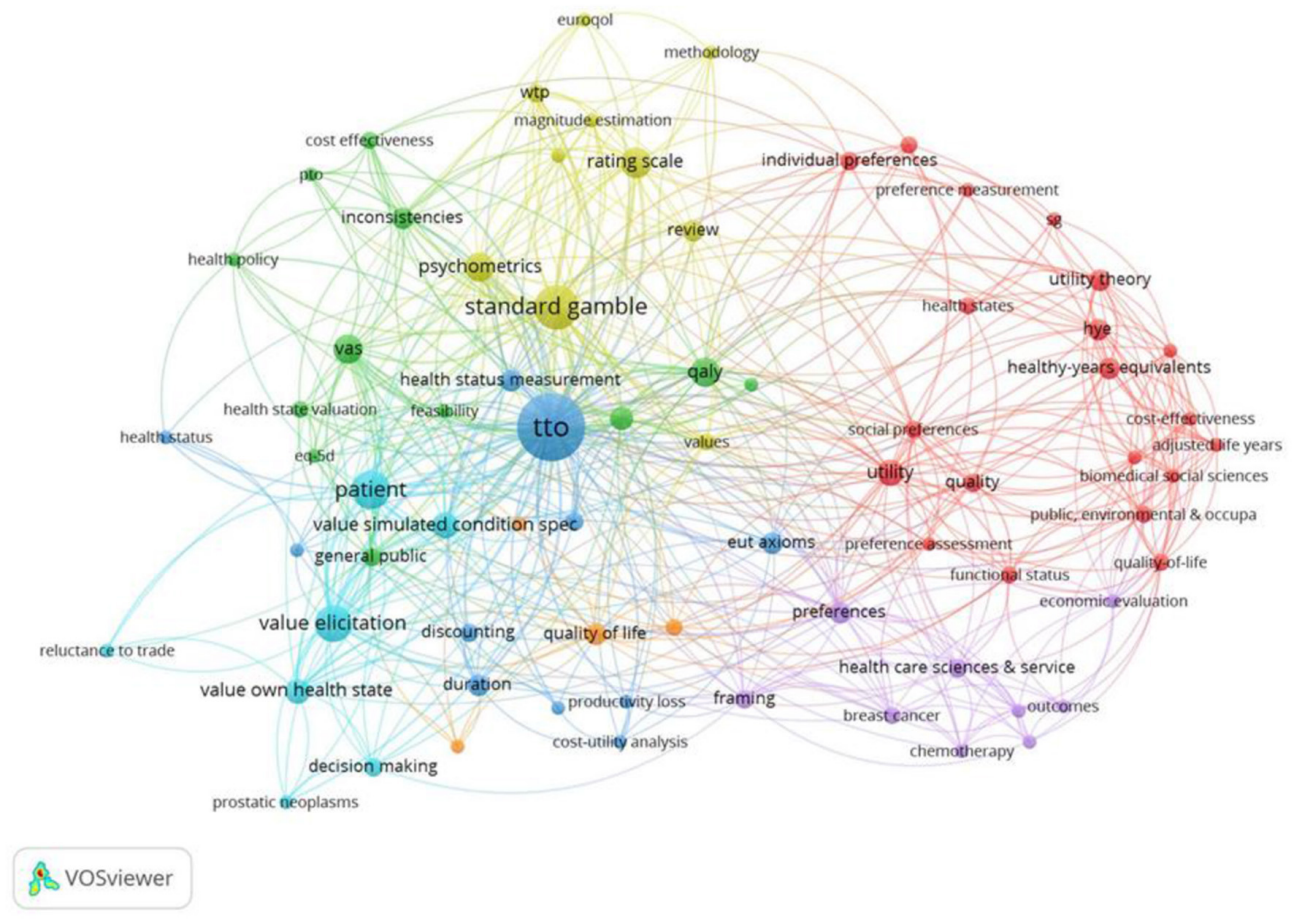
Network visualization for the behavioral set from 1972 to 1999, threshold = 2 (of which 73 keywords met this criteria). PTO, Person Trade Off; EUT axioms, Expected Utility Axioms; VAS, Visual Analog Scale.

The first period covers 70 papers and includes 256 unique keywords. The keywords in Figure 7 include utility theory (55–59), tests of Expected Utility Axioms (60–64), considerations of discounting (65–68), and inconsistencies (69–73). The keyword, “*framing*,” relates to articles considering the framing of the questions in terms of gains or losses (74) and considerations of Prospect Theory to explain both risky and riskless choices (75), as well as the framing of the health state descriptions (76). Additionally, the Healthy Years-Equivalents method (77) was proposed as a method to value multi-state health profiles, and so could accommodate preferences for the order in which health states occurred. This paper preceded the paper by Loewenstein and Prelec (14) that outlined psychological mechanisms that may underpin these preferences. Value elicitation was first mentioned by Torrance (63), but it received little attention until the second half of the time period. Hornberger et al. (78) looked at the variability among methods to assess patients' wellbeing, leading to value elicitation becoming more influential in the early 1990's.

#### Keywords: Period 2000–2010

**Figure 8:**
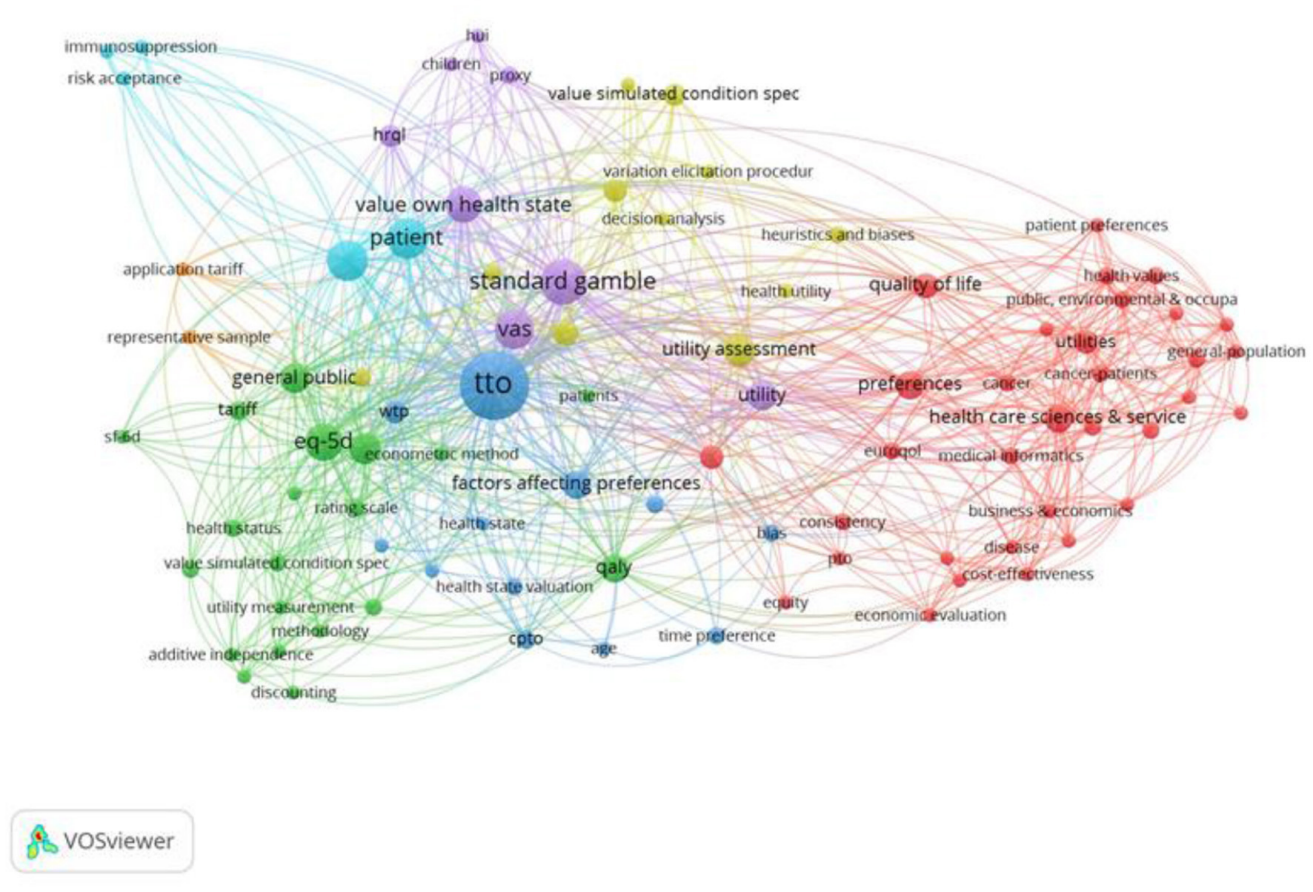
Network visualization for the behavioral set 2000–2010, threshold = 2 (of which 91 keywords met this criteria).

The second set of 70 papers cover the period 2000–2010 and includes 289 keywords, with 73 occurring at least twice, shown in Figure 8. New keywords such as heuristic (79–81) and bias (82, 83) and Prospect Theory (84, 85) are present. In addition, keywords relating to testing Expected Utility axioms, such as Constant Proportional Trade off (CPTO) as well as testing the axioms of additive independence, which is typically imposed alongside expected utility to estimate Quality Adjusted Life Years, or utility independence (86). The review also identified methods, such as the Annual Profile Method (87) that were designed to capture the preferences toward temporary illness within a health profile. The EQ-5D instrument was first introduced late in the first time period (1997) when Dolan first used the EQ-5D TTO tariff. Despite this, its significance can already be seen within the second time period (the green cluster in Figure 8). In Appendix 3 in Supplementary Material, we explore the first and second periods combined, covering the years 1972–2010.

#### Keywords: Period 2010–2015

**Figure 9:**
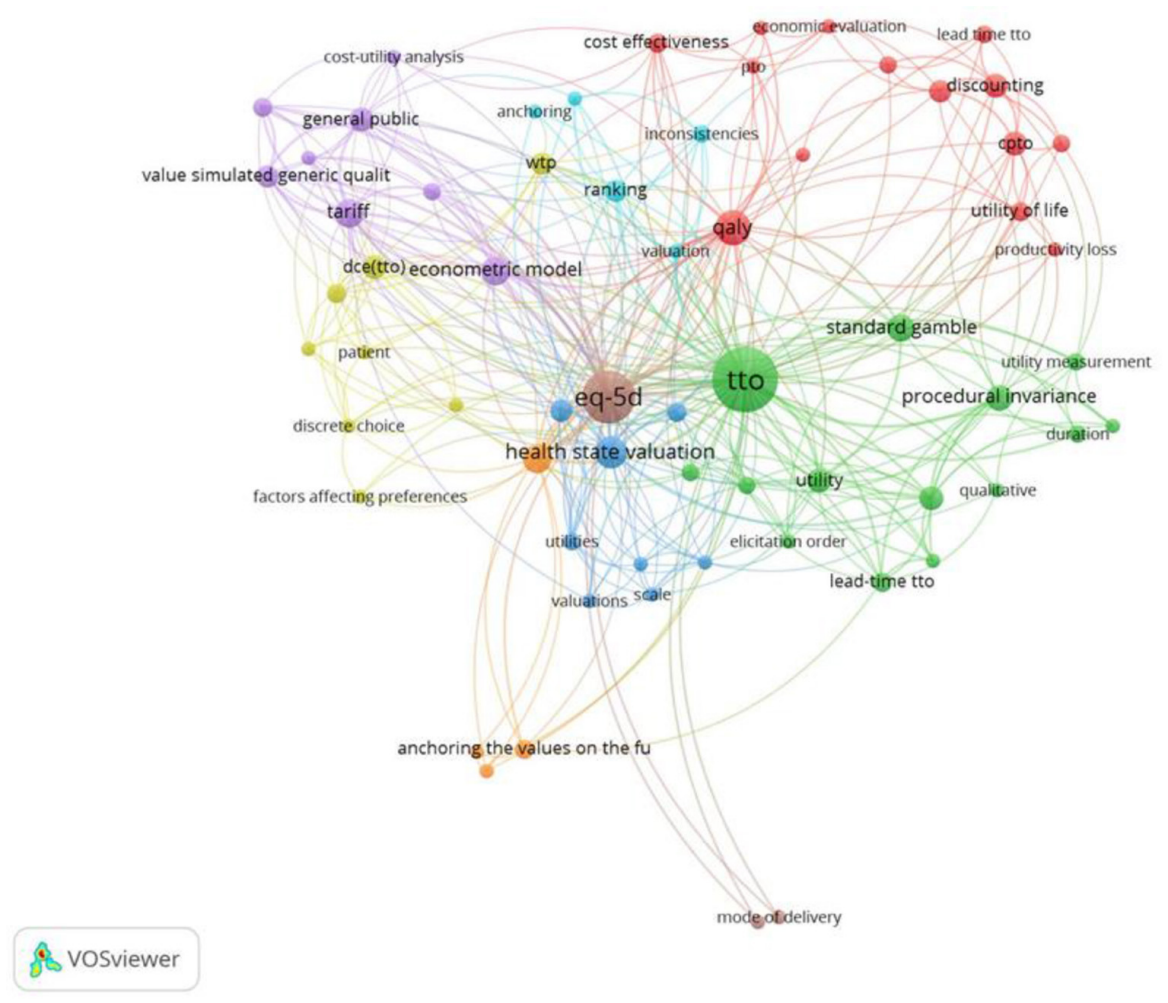
Network visualization for the behavioral set 2010–2015, threshold = 2 (of which 64 keywords met this criteria).

The third set of 70 papers covers the period 2010–2015 and includes 183 keywords, shown in Figure 9. New keywords on further investigations of procedural invariance (6, 10, 88–92) and various elicitation procedures (93–95) are present. Additionally, exploration of methods to elicit states better and worse than dead that standardized the order in which health states (96, 97) such as the Lead Time TTO are discussed (98), to mitigate unintentional Order Effects arising in the health state utility elicitation process. Importantly, new keywords arise on the use of ordinal and/or ranking data to assess preferences. These keywords relate to DCEtto, which was developed to include length of life into the DCE questions (99). Similarly, in the analysis health state valuation (dark blue), this keyword was first mentioned by Dolan (100) and despite initially being important to the TTO literature, faded in the second period, before being reintroduced into the debate by Tilling et al. (97). In Appendix 4 in Supplementary Material, we explore the first, second and third periods combined, covering the period from 1972 to 2015 and 537 keywords.

#### Keywords: Period 2015–2021

**Figure 10:**
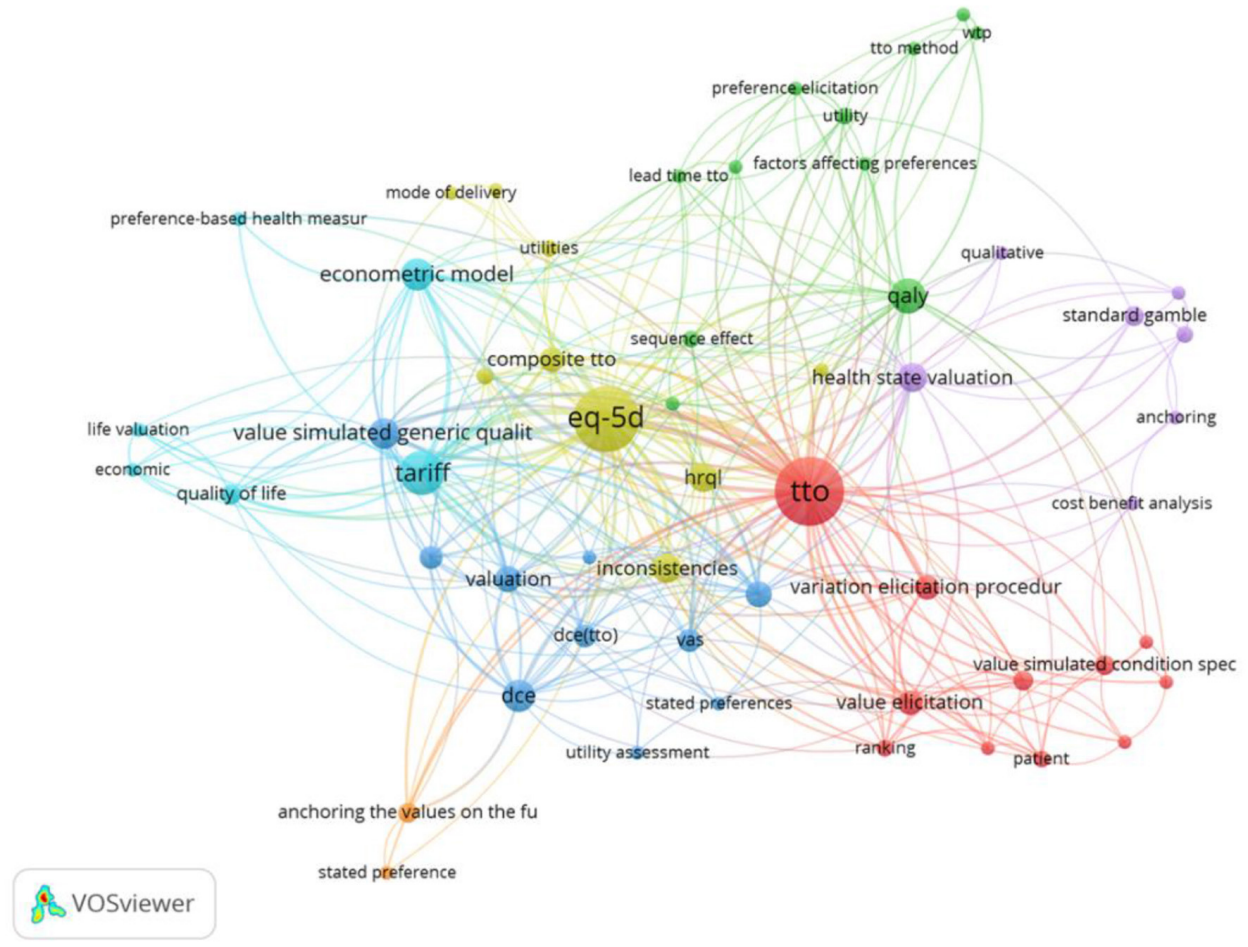
Network visualization of key words of the behavioral set, period 2015–2021, in which 219 keywords appear, threshold = 2 (of which 64 keywords met this criteria).

The fourth set of 70 papers covers the period 2015–2021, shown in Figure 10. New keywords such as composite TTO, that uses a conventional TTO method to value “better than dead states” and the Lead Time TTO to elicit “worse than dead states” are now included (101–106). Importantly, methods are introduced to adjust the TTO values to correct for Non-Expected Utility Theory, shown by the key word “corrective procedures” (107). Additionally, keywords relating to Prospect Theory are now also visible on the map (108). Anchoring the values on the full health and dead scale (orange) is closely associated with DCEtto and ranking. Anchoring is also strongly connected to TTO more generally but in a different sense, possibly related to behavior where the respondents use the value of the first health state as a reference or anchor for subsequent values (109).

#### Keywords: Overview 1972–2021

**Figure 11:**
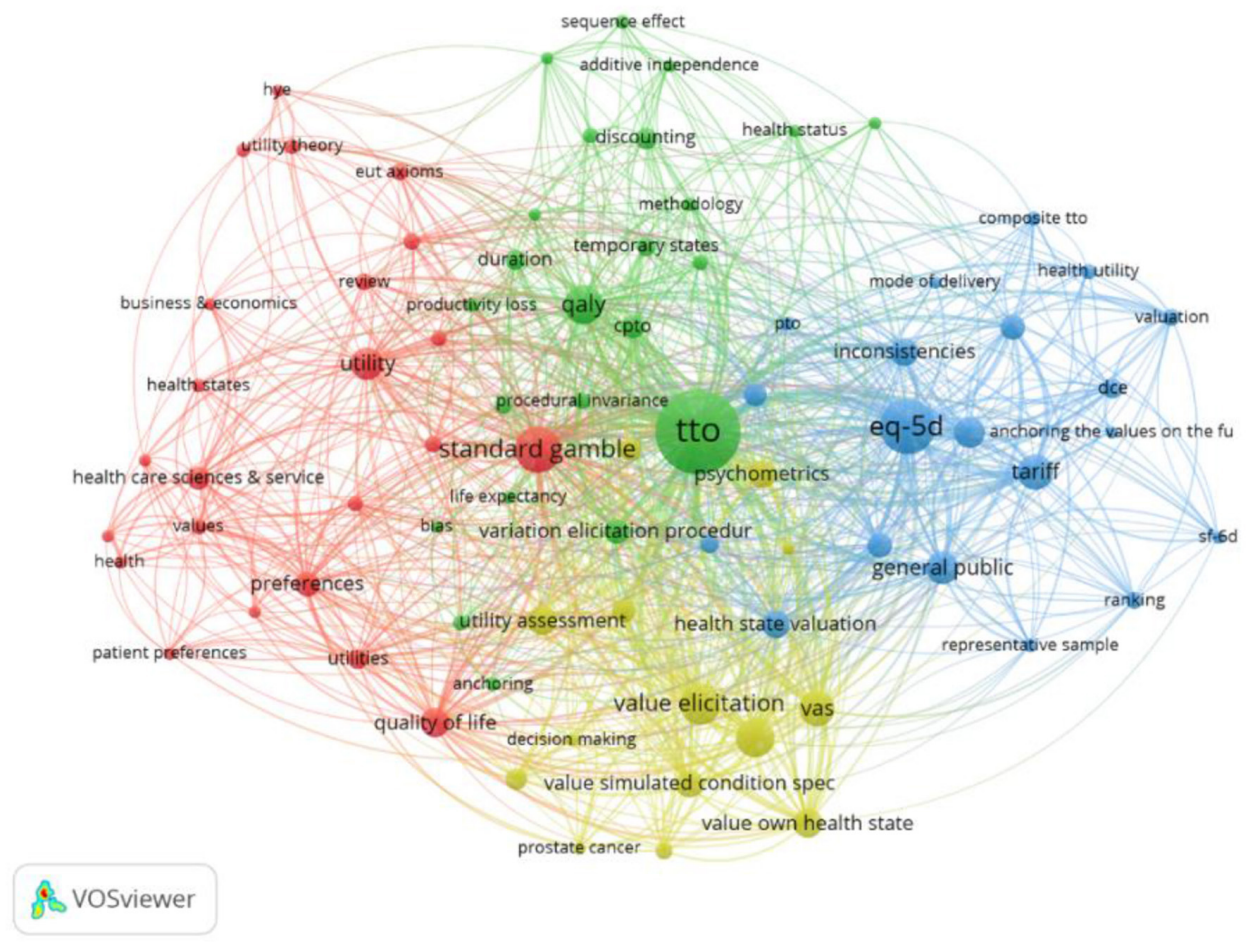
Network visualization of key words for the behavioral set 1972–2021 in which 654 keywords appeared, threshold = 5 (80 keywords met this criteria).

In Figure 11, all four periods are combined, covering the full period from 1972 to 2021. This included a total of 654 keywords. VOSviewer performed a cluster analysis to identify clusters of closely related keywords (54), with related clusters shown in different colors. Figure 11 shows four distinct keyword clusters, based on the number of times they are co-cited, although there is some overlap between clusters. These clusters fall broadly into the following research areas: the lower left area of the visualization display explorations of discounting, additive independence and Non-Expected Utility Theories (green) and link to applications also involving SG and TTO. The lower right area displays keywords relating to Lead time TTO and chaining health states indirectly to full health and death (yellow); the upper right area includes discussion of ranking and choice and the Probabilistic Choice Theory (blue); and the upper left area relates to applications to QALY within cost effectiveness (red). DCE and DCEtto are linked on the map. This is partly because some studies that use DCE with duration do not refer to them as “DCEtto” and had the term “DCEtto” added as reviewer-keywords. On the other hand, DCE (without duration) is linked to the anchoring of the values to full health (=1) and being dead (=0), representing those studies that use DCE (without duration) to value health states on a latent scale and then anchor the values using external data. When the external data for anchoring come from a TTO of one or two states (typically including the worst state), this can be seen as a hybrid use of the TTO alongside DCE that is distinct from its original use to estimate the whole value set.

**Figure 12:**
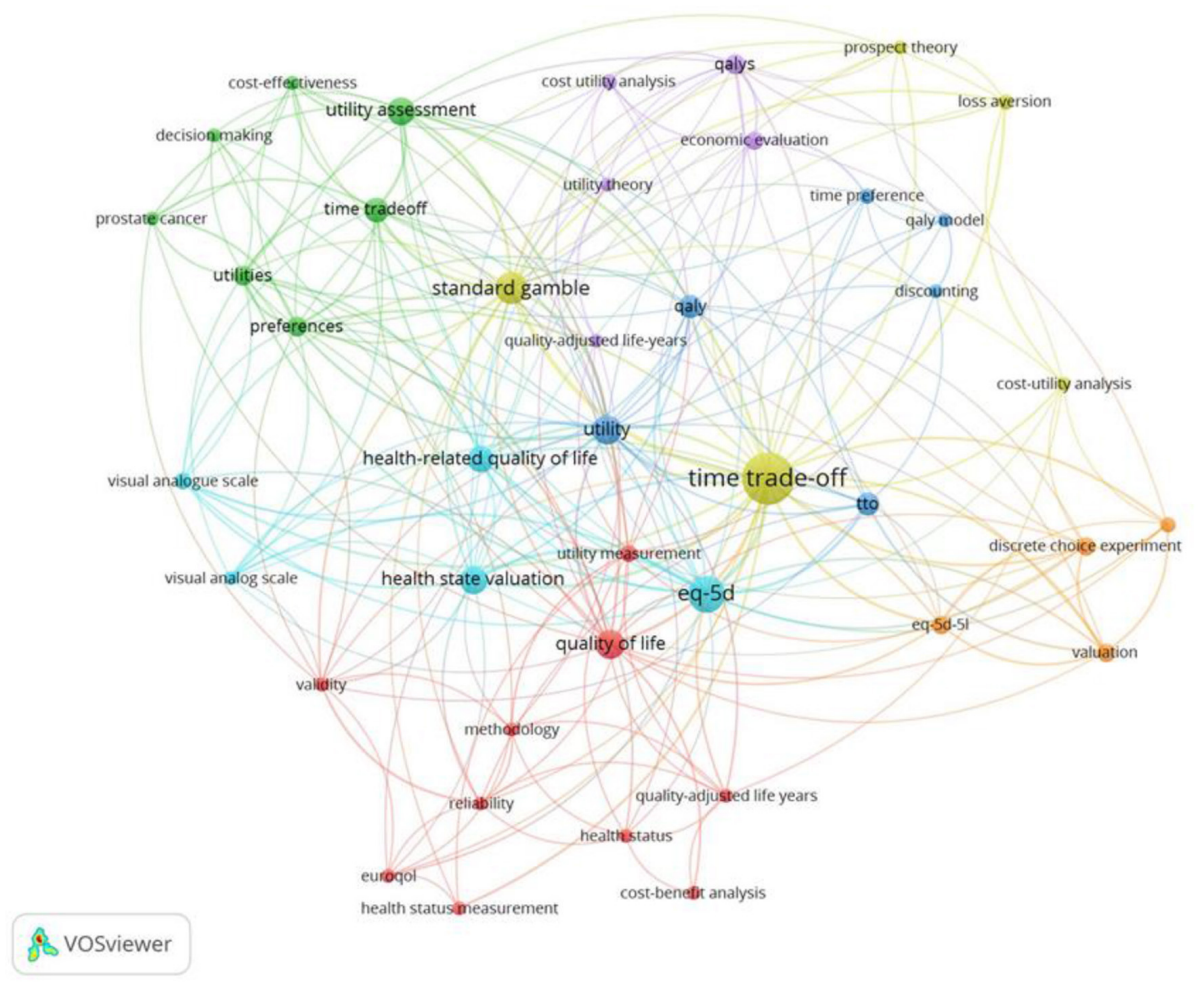
Network visualization of author keywords for the behavioral set of papers (1972–2021) in which 496 author-keywords appeared, threshold = minimum of 5 occurrences (42 keywords met this criteria).

To illustrate the additional value of reviewer-added keywords, Figure 12 shows the author keywords for the behavioral set of papers covering the period 1972 to 2021. There were 496 author keywords, compared to 654 author and reviewer-added terms. Thus, there were 158 new keywords. Of these, 12 were merged, de-duplicated and standardized (e.g., TTO, Time trade off and Time-trade off merged), and a further 146 were added according to the categorization (Appendix 1 in Supplementary Material). For consistency, a threshold of a minimum of 5 occurrences was used in both analyses that cover the full period of the study.

## Discussion

Our study explored the use of data weaving, a combination of scoping review, bibliometrics and visualization tools to conduct temporal, geographical, and key word analysis (43). The study highlighted that not only did the Netherlands, UK and US have the most publications but the international co-authorship was predominantly between/with the UK, US and Netherlands. The UK and Netherlands have consistently published at the forefront of the methodological developments over the entire time period and the analysis conducted suggests this is unlikely to change soon.

The review additionally set out to develop an understanding of how behavioral theories have influenced the way preferences for health-related quality of life are elicited and interpreted over time. We ordered the papers chronologically and split them into four sets of equal size, to explore the uptake in the theories over time into the TTO method. In this section we return to this question and explore the uptake of these theories over time.

## Expected Utility and Non-Expected Utility Theory

Although the TTO used in health state valuation is attributed to Fanshel and Bush (7), and Torrance et al. (8), it was not until Pliskin et al. (12) that the axiomatic basis was developed using Expected Utility Theory (33). This review did not identify any earlier paper drawing upon Expected Utility Theory within health state preference elicitation. The introduction of Non-Expected Utility Theory (110) came about through a series of papers that drew on Prospect Theory to provide additional explanations for systematic differences between the SG and TTO values. However, this review did identify additional early papers that described the importance of Prospect Theory in health state preferences, such as Stalmeier and Bezembinde (75), as well as more well-known papers by Treadwell (17) and Bleichrodt (85) and failures of procedural invariance in the TTO method by Spencer (111) ascribed to Prospect Theory. This incorporation of Prospect Theory into health state elicitation led to further work around the predictive validity of Prospect Theory (112, 113). More recently, Lipman et al. (107) proposed a non-parametric approach to simultaneously correct for loss aversion, probability weighting and non-linear utility of life duration.

## Order Effects

Establishing the existence of preferences over the order or sequence in which events occur over time were preceded by Healthy-Years Equivalents method (58) which valued sequences of health states. This review highlighted a number of papers looking at the limitations imposed by assuming that utilities from constituent health states can be combined over time, ignoring how health varies over time from constituent health states when health varies over time (16, 17). Research methods motivated by these concerns have been used to value temporary or recurring states to ensure Order Effects are captured, but the values thus derived are not routinely drawn upon for policymaking (114). The review identified methods, such as the Annual Profile Method (87) that were designed to capture the preferences toward temporary illness within a health profile but that did not involve the two-step procedure of Healthy Years-Equivalents which has come under criticism. The review also explored considerations that elicitation procedures may unintentionally introduce Order Effects, for example, by changing the sequence in which full health appears in the profiles used to value better than dead and worse than dead states and a proposed method to standardize the elicitation procedures to eliminate such confounding (96). Devlin et al. (115) termed this standardized procedure the Lead time TTO method (115), and they are still used in the composite TTO methodology to value EQ-5D-5L. However, the additive separability that was assumed by these methods has since been shown to be violated by interaction effects with earlier periods (10, 116). Elicitation procedures may also introduce Order Effects through the order in which health states are valued, and some studies referring to anchoring in TTO may be referring to this Attema et al. (18) and Chuang and Kind (117).

## Probabilistic Choice Theory

Probabilistic Choice Theory superseded deterministic choice and led to DCEs (20) being used to elicit preferences. DCEs were used to value health states, and the ordinal scale was then linked to a cardinal scale with 1 for full health and 0 for being dead initially using an external anchor, such as a traditional TTO question for a chaining state (118). The review confirmed that it was not until Bansback et al. (99) that the DCEtto was developed which included length of life into the DCE questions to anchor the latent parameters on the full health to dead scale. Equivalent explorations for the SG method, including risk into the DCE questions, allow estimation of probability weighting functions and the valuation of a state worse than dead (119).

## Limitations

### Research Scope and the QALY

The study focused upon the TTO method given it represents the QALY concept—that survival in less than full health can be deemed equivalent to a shorter survival in full health. In addition, the TTO underpins Health Technology Assessments that use the QALY approach. It could be argued that other preference elicitation methods, such as the SG incorporate additional aspects such as preferences toward risk and uncertainty and reflect relevant behavioral theories arising from Economics. While the papers identified in this review include those that had discussed the SG method alongside the TTO method, it is likely to have missed papers focusing exclusively on the SG. Both the SG (120) and TTO (121) were initially explored in the UK valuation set of the EQ-5D-3L, but the SG was quickly dropped in favor of the TTO (122). Later, resurgence of the SG using choice-based methods and allowing valuing of states worse than dead in the same framework have not been taken up (119).

The review identified Dolan 1997 as a highly cited paper, which raised questions about whether Dolan 1997 should have been a source paper. Either way, this presents an interesting consideration as to the choice and timing of source papers. For example, should it be based on an initial review on the same topic? The issue of source papers is more likely to affect reviews based solely on citation of source papers, such as the review conducted by Spencer et al. (30), and less so for our paper that is based on a systematic review of articles that included the term time-trade off.

We are aware that many of the authors appearing in Figure 3 are (or were) members of the EuroQol group (https://euroqol.org/). The group not only owns and controls the EQ-5D instrument, but funds scientific research. While members of this group have had a substantial impact on the TTO literature since the 1990s, exploring EuroQol Group membership of individual authors and EuroQol Group research funding of specific empirical projects was beyond the remit of the review. Regarding the history of the EuroQol Group, the interested reader is referred to Kind et al. (123) and Brooks (124).

### Broader Issues Around the Natural Life Cycle of Papers and Self-Citation

A potential limitation of co-citation analysis is that the publication date may affect the number of citations that a paper receives. For example, it is more likely that recent articles have received insufficient citations to be included in the analysis whilst for older papers, citations may decline as the innovative aspects are reflected in later papers or large-scale reviews. These ideas suggest that there is likely to be a natural life cycle of a paper with citations initially increasing then decreasing over time, which creates challenges in how to interpret citations over time.

It is also possible that self-citation accounts for some of the results of the citation analysis and may bias interpretation. There is no functionality in VOSviewer that would allow the users to exclude self-citation in maps generated by the tool.

### Functionality of Mapping Techniques

The VOSviewer software allows for a vast amount of information to be analyzed quickly and enables the creation of intricate maps. As the number of citations increase, it is necessary to impose inclusion thresholds for the visuals to reduce the complexity. Whilst the thresholds applied throughout the visualizations were low (1–5 citations), we were unable to display all the nodes in the two-dimensional space. There is some judgement about thresholds to impose but changing the threshold does not change the source data. To allow others to explore these visuals with other thresholds we have included an online data repository (Appendix 4 in Supplementary Material).

It should also be noted that VOSviewer provides a range of mapping tools, but care and judgement is needed on how to apply these to facilitate interpretation. For example, we found the time dimensions graphics of VOSviewer was hard to interpret in our review, as the shadings assigned represented the average of the time period covered (so if the citations covered 2010–2020, the average of 2015 would be assigned). In our study we aimed to overcome this limitation by ordering papers chronologically and splitting them into four sets of equal size, to explore the influence of the behavioral theories on the TTO method. Other approaches may also be possible, for example, by considering changes in the themes relative to a baseline. However, in our initial explorations, we found alternative visualizations hard to operationalize due the inevitable need to change the thresholds citations to accommodate larger maps.

A final drawback is that VOSviewer is unable to differentiate between various spellings of the same author's name, such as when initials are used. The analysis could therefore be potentially affected by VOSviewer's inability to recognize and disambiguate the author/journal names in the visualizations created from the bibliometric data (125). Thus, additional steps had to be taken to ensure authors were credited with the correct number of articles. In our study, we manually and repeatedly saved cited references in endnote and citations from Scopus, but this process is time-consuming and cumbersome to complete and limits the user-friendliness of VOSviewer.

### Challenges to the Application and Interpretation of Keyword Analysis

In our study, reviewer-added keywords were used to summarize the contribution captured in the title or abstract, and the cluster analysis implemented was at a keyword level, to allow conclusions to be drawn about the research questions or topics. However, reviewer-added keywords take time and require in-depth knowledge of the topic area. An automated process, such as “keywords plus,” was suggested by Zhang et al. (51) and may provide advantages in terms of processing time, provided the use of keywords is stable over time and reliable across researchers.

Perhaps a greater challenge to the application of keywords analysis was the ability to discern the uptake of behavioral theories. The visuals provide a good summary of individual keywords, and can show the appearance of new or changing keywords, but individual keywords alone, nor network of keywords, cannot easily summarize theory uptake. There is also no facility within VOSviewer to run keyword analysis alongside a paper-level analysis. Instead, we found it necessary to link the keywords back to the source papers manually in order to understand the context in which these keywords were used and the underlying papers. In addition, the keyword analysis groups papers by terms, and these may not have a one-to-one link to the concepts behind them. The same term may have subtly different meanings, or the same concept may be represented by different terms, and these may change both over time and across authors. The keyword visuals, therefore, provide a high-level view only. This high-level view works well for simple analyses where the method is encapsulated succinctly within keywords, such as uptake of specific methods, such as SG, TTO or VAS. However, in more nuanced analyses, as we undertook here, it becomes increasingly important to link back to the source papers.

### Future Research

We recommend that future research extends the analysis to other methods such as SG, to explore how this has responded to and taken up these behavioral theories over time. The systematic searching of both SG and TTO papers would have increased the burden on the manual inclusions/exclusion criteria and was beyond resources of the current study here. Future research could also consider further applications of data weaving by applying automated systems to classify paper content, using such systems as data mining to identify the main contribution of papers. It could also utilize alternative procedures to explore visualizations over time, alongside other complementary methods, including multivariate trend analysis for authors that have published multiple papers over time, to help tease out the intra-evolution of ideas. Finally, we recommend research into how to explore literature developments over time that considers the natural life cycle of papers. A smaller review looking at relatively few papers, or a sample of the behavioral set, could also be conducted to assess the role of self-citation. For example, in Scopus, it would be possible to take one or more documents and run an overview of citations for the 280 behavioral set: i.e. find out the total number of citations to these papers and then exclude the self-citations to find out the proportion of self-citations but this is beyond the scope of this study.

## Conclusion

This paper represents one of the first applications of data weaving in health economics to understand the methodological theories influencing the literature on the QALY and TTO methodology. Applying this form of analysis to health state valuation has the potential to enrich our understanding of the developments in the TTO method and suggest further work. The method was particularly successful in summarizing high-level themes and connections. For example, VOSviewer evidenced the most influential contributors to the literature at the author, paper, and country levels whilst keywords analysis highlighted high-level themes, such as the specific axioms that were tested. In some instances, the approach additionally identified papers that were contributing to the debate. Though these high-level views are helpful: they complement the systematic review rather than stand alone, as the underlying papers are required for interpretation of the keyword analysis. In the discussion we reflect on this and other limitations and identify aspects that should be considered when applying this method in future reviews for health economics and social sciences.

It has been argued elsewhere that many studies that elicit TTO value sets have failed to engage with some of the recent behavioral developments. Many studies still assume Expected Utility Theory and interpret the values without considering the implications of Non-Expected Utility models or failure of constant discounting. Our paper highlights the delay in uptake of the four behavioral theories into the practice of health state valuation. International protocols, for example for the valuation of EQ-5D-5L (126), are one way to guide how the TTO is implemented and interpreted by standardizing procedures and have been effective at minimizing anomalies due to Order Effects. However, we would argue that these protocols do not go far enough to engage with the application of Non-Expected Utility Models (107). It is therefore important that researchers within health economics work more closely with those in mainstream economics and keep abreast of the wider economics and behavioral sciences to expedite the uptake of new ideas.

## Author Contributions

LH, AS, RW, and AT contributed to the development and design of the study. LH, RW, and AS performed the statistical analysis and coding of keywords. LH and AS wrote the first draft and the final manuscript of the manuscript. All authors contributed to manuscript revision, read, and approved the submitted version.

## Conflict of Interest

The authors declare that the research was conducted in the absence of any commercial or financial relationships that could be construed as a potential conflict of interest.

## Publisher's Note

All claims expressed in this article are solely those of the authors and do not necessarily represent those of their affiliated organizations, or those of the publisher, the editors and the reviewers. Any product that may be evaluated in this article, or claim that may be made by its manufacturer, is not guaranteed or endorsed by the publisher.
