# Supplementary material for: Behavioral Theories That Have Influenced the Way Health State Preferences Are Elicited and Interpreted: A Bibliometric Mapping Analysis of the Time Trade-Off Method With VOSviewer Visualization
Source: Front Health Serv. 2022 Jul 1;2:848087. doi: 10.3389/frhs.2022.848087 (PMC10012726; doi:10.3389/frhs.2022.848087)
Supplement: Supplementary file 1 [file Data_Sheet_1.pdf]

Appendices

Appendix 1: Keywork categorisation
(merged terms in red, reviewer added keywords in purple).

**Expected utility**	
TTO axioms	CPTO (merge, Constant proportional Time Trade-off, CPTO)<br>MET (merge anything with: Maximal endurable time, MET), transitivity, EUT axioms (merge: Expected Utility, von Newman Morgenstern Utility, axioms)
Discounting	Stationarity (merge hyperbolic discounting, impatience, increasing impatience), time inconsistencies, discounting (merge: Double discounting, discount, discount rates), time utility independence
Utility of Life	Utility of life (merge utility of life duration, utility curvature, utility function for survival time, diminishing marginal disutility, decreasing marginal value of life, risks in life duration
Life expectancy	Life expectancy (merge: life expectancy, subjective life expectancy),
Aggregation across individuals	interpersonal comparisons of utility (merge: interpersonal, interpersonal comparisons of utility
Aggregation across domains	Correlation aversion, productivity loss (productivity loss, income, consumption activities), life stage (carer v non carers, life goals)<br>Altruistic preferences in time tradeoff
**Non expected utility**	
Non expected utility models	Prospect Theory, probability weighting, loss aversion, regret theory (merge regret, regret theory), corrective approach , reference point, corrective for discounting
inconsistencies	Inconsistencies (merge health state valuation inconsistency, logical inconsistencies, utility inconsistencies, time inconsistency)
**Procedural invariance**	
Biases and procedural invariance	Anchoring (merge: anchoring, anchoring bias, reference point, starting value, Anchoring and adjustment bias), heuristics and biases (recall bias, Proportional heuristic), context effects, preference reversals (merge common ratio effect, preference reversal), scope insensitivity, Scale compatibility, magnitude effect, framing (merge: framing effects, framing), learning effects, procedural invariance (merge procedural invariance, iteration bias), construct preferences
Within elicitation procedures procedural invariance	iteration (merge: iteration, titration, ping pong), elicitation order (merge elicitation order, sampling EQ-5D states)
chaining	Chained procedure (merge: Chained, chained procedure, chaining),
imprecision	Imprecision preferences

**Ordering of states**	
Order of states	Additive independence (merge: additive independence assumption, additive separability, separability)<br>Sequence effect (merge sequence, sequence effect, sequence effects, diminishing marginal disutility)<br>Health profiles (merge health profiles, profiles, lifetime health profiles)<br>Duration<br>Temporary states (merge: short-term health-state, Temporary health states, health state duration, short-term health conditions)<br>Adaptation
Valuing worse than death states on same scale as better than death scale	States worse than dead (merge: Worse than death state, states worse than dead, states worse than death)<br>Lead time TTO<br>Lag time TTO<br>Composite TTO
**Use of methods**	
Uses of methods	Risk-Benefit (merge risk and benefit, risk-benefit), Cost Benefit Analysis (merge Cost Benefit Analysis, CBA), QALY (merge Quality Adjust life Years, QALY, QALYs), cost effectiveness (merge: cost effectiveness, cost-effectiveness study, cost-utility, incremental cost effectiveness analysis, comparative effectiveness, cost-effectiveness), Oregon, burden of disease, **value elicitation (merge utility elicitation, valuation elicitation, value elicitation etc**.), mapping (mapping, crosswalk), tariff (merge: tariff, index, EQ-5D index, value set, health status index), decision analytic model (merge: markov, decision analytic), application tariff, review utilities, systematic review, application study values, international comparisons (cross-country comparisons), variation of tariff weighting, feasibility, correlation clinical outcome, values used for prediction, personal injury
**Development Tariff**	
Econometric model	Merge (Econometric model, Econometric modelling, econometrics)
frameworks	Compensating variations
**Measurement properties**	
Psychometrics	Psychometrics (merge: Validity, validation, Sensitivity, Lower bound, ceiling threshold, Reliability, construct validity), concordance other clinical scales
Qualitative methods	Thematic analysis<br>Think aloud<br>Mixed method<br>Qualitative methods (merge: MACBETH, qualitative methods, qualitative analysis)<br>Mixed methods
**Random Utility Theory and Choice**	

	DCE (merge: Discrete Choice Experiments, DCE, discrete choice methodology)<br>Scalability<br>Conjoint analysis<br>anchoring the values on the full health-dead scale (merge any related wording to this)<br>Best worse
**Elicitation methods**	
Elicitation methods	TTO (time trade-off technique)<br>TTO long-term care<br>Converted TTO<br>Daily TTO<br>TTO delay (merge: TTO delay, Waiting time TTO<br>sleep trade off<br>Experienced-based valuation<br>SG<br>SG blind (SG blindness, SG trading blind)<br>VAS<br>Annual profile method<br>WTP (merge, contingent valuation, Willingness-to-pa)<br>Ranking<br>Paired comparison<br>Rating Scale (RS)<br>DCE<br>Healthy Years-Equivalent (HYE) (merge: healthy years equivalent, HYEs, etc)<br>Conjoint analysis<br>PTO (merge PTO, Person trade-off etc. Disability Adjusted Life Years (DALY)<br>probability time trade-off (intertemporal risk-value model?)<br>composite TTO<br>risk-risk trade off<br>MAUT (merge Multi attribute utility, Multi attribute utility theory (MAUT))<br>CORE 6D<br>OAB-5D<br>Visual Function Questionnaire-Utility Index<br>NEWQOL-6D<br>8 SBS-QoL™<br>EORTC-8D<br>15D<br>Quality of Life Questionnaire and Neurogenic Module<br>VisQol<br>Chained approach<br>TTO, VAS, WTP<br>Location-of-dead (LOD) method, part of the personal utility function (PUF) approach ,<br>Multicriteria Decision Analysis (MCDA)
Explorations of the procedure to elicit values	individual v collective decision making , variation elicitation procedure (feedback mechanism (, collective

	responses, individual responses, modified method), variation elicitation procedure, variation warm up), online versus face-to-face, anchor disease free or perfect health, sampling EQ-5D states, asked to include income.
Domains covered	Process utility (merge: process of care, process utility, process, treatment process utility), experienced-based preferences, non-use value, well-being, HRQL (merge health related quality of life, HRQL), end of life (merge: End-of-life preferences; end of life), condition specific (merge disease specific, condition specific), treatment delivery (diagnosis preferences, treatment strategies, management strategies)<br>(merge: condition specific medication, treatment delivery), WALY (animal equivalent of DALY), generic QoL (QoL for adolesents, development descriptive system), variation domains covered
Who asked?	Patient (patient preferences), Children (child), general public (merge: general population , general public, community, etc), adult naïve observers, national representative sample (nationally representative), physician (clinician, clinician preferences), Elderly (merge elderly, frail elderly etc.) , carer (merge: carer, caregiver), proxy, student (merge: student, medical student), parent, women, convenient sample, pregnant women, health professionals, adolescent, experts, veterinarian, variation question frame (parent, child, adult) crowd sourcing, convenient sample, family, employees, academics, international, professionals, patient control.
What do they value?	value own health state,<br>value observed patient health state,<br>value experienced treatment delivery,<br>value simulated condition specific health state,<br>value simulated generic quality of life (EQ-5D, tariff etc)<br>simulation patient experience<br>management strategies<br>(merge: condition specific medication, treatment delivery)<br>Screening<br>value simulated data collection, other<br>value simulated health risk redutions, other<br><br>comorbidities/disease

Factors affecting preferences	Factors affecting preferences, attitudes, religion (life after death), age, imprecision (merge imprecise, imprecision), socioeconomic (merge: socio-economic, socioeconomic), age, ethnicity, socioeconomic, societal, life stage, pivotal life event, order of dimensions, cultural, Happiness versus preferences, preference Quality or longevity, adaptation , reluctance to trade, outlook, non-corresponding description.
**Valuation of QALY**	
Valuation of a QALY	WTP for QALY
**Descriptive system**	
Classification systems	EQ-5D, Short form (SF-36, SF-12), SF-6D, Ascot, condition specific,<br>multi attribute (multiattribute utility theory), HUI (merge HUI, HUI2, HU13), CHU9D (Child Health Utility 9D), VisQoL, Utility-Based Questionnaire-Cancer, EORTC QLQ-C30
Adding dimension	Adding dimension (merge: extra dimension, extra dimension in health state description), Bolt-on (merge bolt-on, Bolt on, EQ-5D + C)), adjusting dimension
Comorbidity, disease burden	Aggregation comorbidity (merge: Aggregation comorbidity, multiple conditions (Meltzer's paper, extra dimension), joint health states
Additional Factors	Labelling (merge any with label, labelling),<br>Description of dead
**Broad terms**	
Time preference	Time preference, time horizon, intertemporal,<br>Years of life lost
Risk preference	Time preference, risk preference, equity (altruism), time horizon, intertemporal, risk attitude, risk,<br>Years of life lost, heterogeneity, societal, uncertainty, age, ethnicity, socioeconomic, societal<br><br>Some are merged, e.g. risk and inequality aversion
equity	equity (altruism)
**Mode of delivery**	
	Mode of delivery (merge: Interview, online, face-to-face, face-to-face versus online)

Appendix 2: network analysis of the co-occurrence of keywords, combining the first 70 papers (1972-1999) with the second period (2000-2010), representing 140 articles (Threshold = 3, 91 keywords met this).

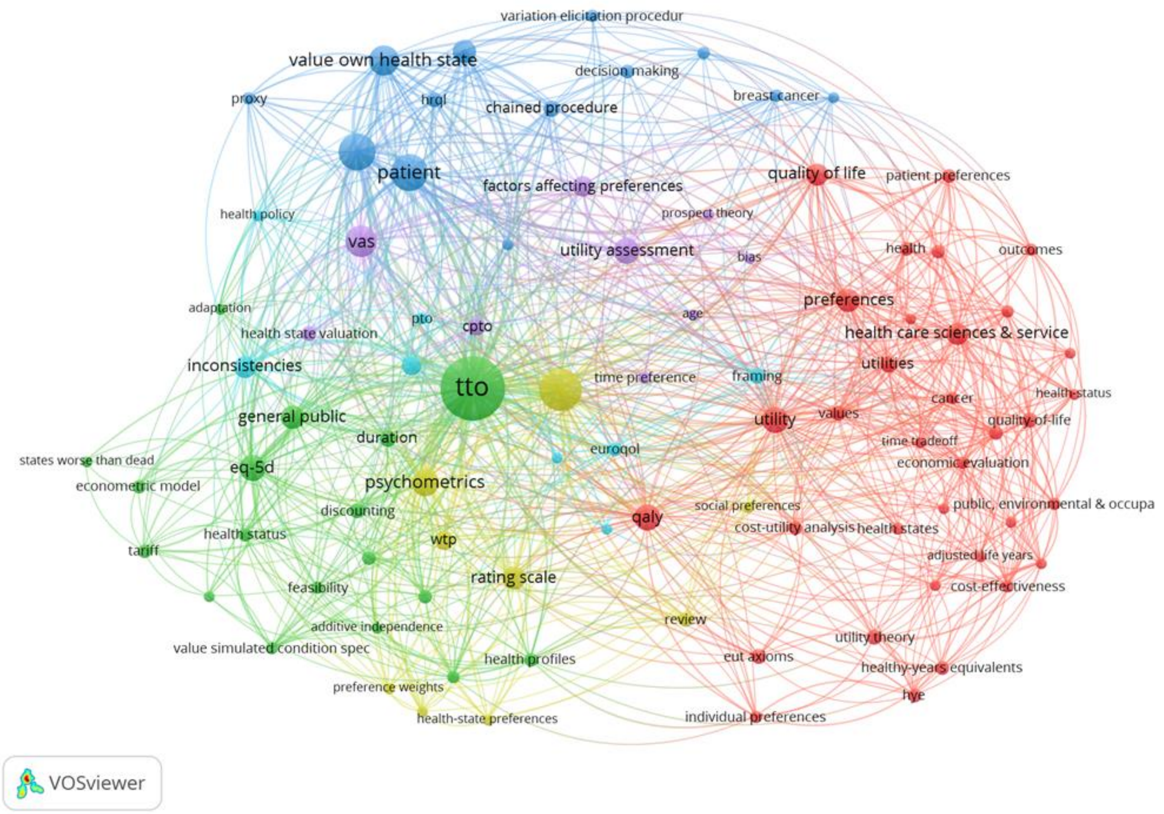

Appendix 3: network analysis of the co-occurrence of keywords, combining the first 140 papers (1972-2010) with the next 70 papers (2010-2015), representing 210 articles (Threshold = 4, 87 keywords met this).

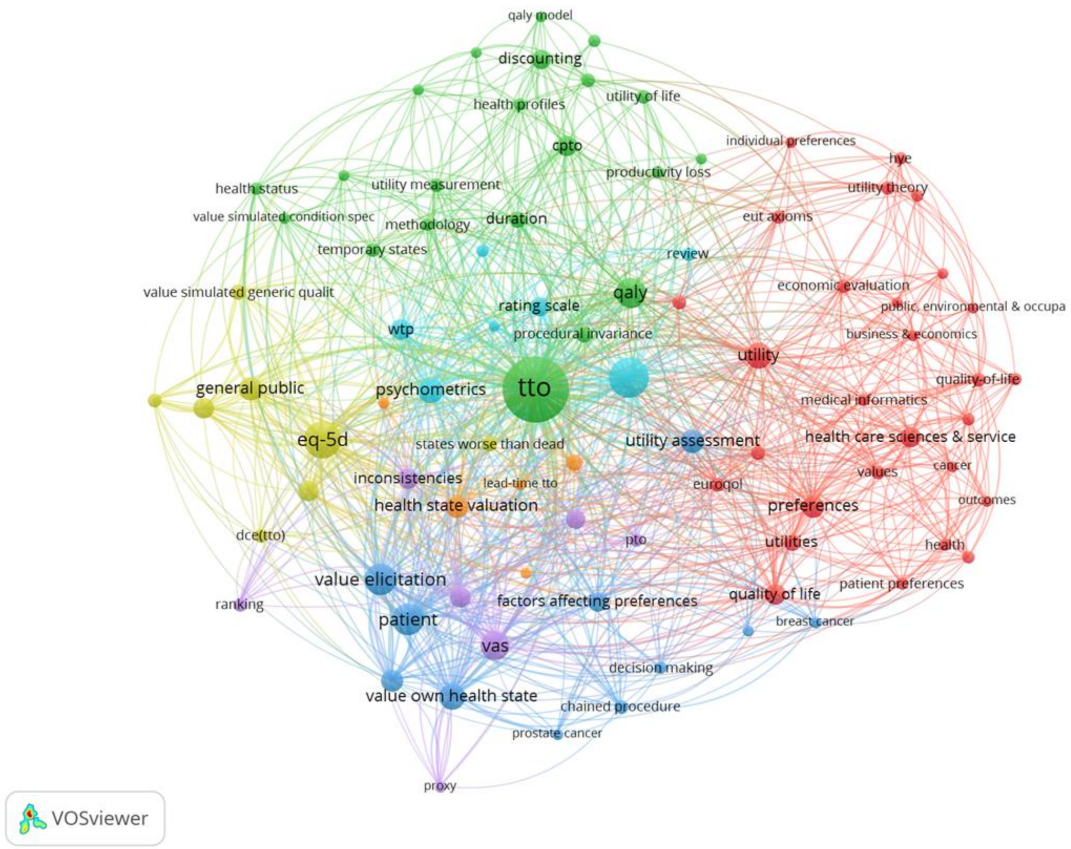

Appendix 4: The full list of papers included in the keyword, authorship, co-authorship analysis of the behavioural set.

Period 1

Author	Year	Title
S. B. Goldsmith	1972	STATUS OF HEALTH STATUS INDICATORS
J. S. Pliskin, D. S. Shepard and M. C. Weinstein	1980	UTILITY-FUNCTIONS FOR LIFE YEARS AND HEALTH-STATUS
G. W. Torrance	1982	Preferences for health states: a review of measurement methods
G. W. Torrance, M. H. Boyle and S. P. Horwood	1982	APPLICATION OF MULTI-ATTRIBUTE UTILITY-THEORY TO MEASURE SOCIAL PREFERENCES FOR HEALTH STATES
H. Llewellynthomas, H. J. Sutherland, R. Tibshirani, A. Ciampi, J. E. Till and N. F. Boyd	1984	DESCRIBING HEALTH STATES - METHODOLOGIC ISSUES IN OBTAINING VALUES FOR HEALTH STATES
G. W. Torrance	1986	Measurement of health state utilities for economic appraisal. A review
G. W. Torrance	1986	MEASUREMENT OF HEALTH STATE UTILITIES FOR ECONOMIC APPRAISAL - A REVIEW
G. W. Torrance	1987	Utility approach to measuring health-related quality of life
D. G. Froberg and R. L. Kane	1989	Methodology for measuring health-state preferences-II: Scaling methods
A. Mehrez and A. Gafni	1990	EVALUATING HEALTH RELATED QUALITY-OF-LIFE - AN INDIFFERENCE CURVE INTERPRETATION FOR THE TIME TRADE-OFF TECHNIQUE
D. C. Hadorn	1991	THE ROLE OF PUBLIC VALUES IN SETTING HEALTH-CARE PRIORITIES
J. C. Hornberger, D. A. Redelmeier and J. Petersen	1992	Variability among methods to assess patients' well-being and consequent effect on a cost-effectiveness analysis
E. Nord	1992	Methods for quality adjustment of life years
K. Gerard, M. Dobson and J. Hall	1993	FRAMING AND LABELING EFFECTS IN HEALTH DESCRIPTIONS - QUALITY ADJUSTED LIFE YEARS FOR TREATMENT OF BREAST-CANCER
M. Johannesson, J. S. Pliskin and M. C. Weinstein	1993	ARE HEALTHY-YEARS EQUIVALENTS AN IMPROVEMENT OVER QUALITY-ADJUSTED LIFE YEARS

R. M. Kaplan, D. Feeny and D. A. Revicki	1993	Methods for assessing relative importance in preference based outcome measures
H. A. Llewellyn-Thomas, H. J. Sutherland and E. C. Thiel	1993	Do patients' evaluations of a future health state change when they actually enter that state?
A. Mehrez and A. Gafni	1993	Healthy-years Equivalents versus Quality-adjusted Life Years: In Pursuit of Progress
J. Tsevat, L. Goldman, J. R. Soukup, G. A. Lamas, K. F. Connors, C. C. Chapin and T. H. Lee	1993	STABILITY OF TIME-TRADEOFF UTILITIES IN SURVIVORS OF MYOCARDIAL-INFARCTION
M. Johannesson	1994	QALYs, HYEs and individual preferences- A graphical illustration
M. Johannesson, J. S. Pliskin and M. C. Weinstein	1994	A Note on QALYs, Time Tradeoff, and Discounting
A. M. Stiggelbout, G. M. Kiebert, J. Kievit, J. W. H. Leer, G. Stoter and J. C. J. M. de Haes	1994	Utility Assessment in Cancer Patients: Adjustment of Time Tradeoff Scores for the Utility of Life Years and Comparison with Standard Gamble Scores
A. M. Stiggelbout, G. M. Kiebert, J. Kievit, J. W. H. Leer, G. Stoter and J. Dehaes	1994	UTILITY-ASSESSMENT IN CANCER-PATIENTS - ADJUSTMENT OR TIME TRADEOFF SCORES FAR THE UTILITY OF LIFE YEARS AND COMPARISON WITH STANDARD GAMBLE SCORES
P. Dolan and C. Gudex	1995	Time preference, duration and health state valuations
F. J. Fowler, Jr., P. D. Cleary, M. P. Massagli, J. Weissman and A. Epstein	1995	The Role of Reluctance to Give Up life in the Measurement of the Values of Health states
A. Gafni and S. Birch	1995	PREFERENCES FOR OUTCOMES IN ECONOMIC-EVALUATION - AN ECONOMIC-APPROACH TO ADDRESSING ECONOMIC-PROBLEMS
J. F. O'Leary, M. K. Jankowski and J. C. Weeks	1995	Comparison of Time-tradeoff Utilities and Rating Scale Values of Cancer Patients and Their Relatives: Evidence for a Possible Plateau Relationship
A. M. Stiggelbout, G. M. Kiebert, J. Kievit, J. W. H. Leer, J. D. F. Habbema and J. C. J. M. De Haes	1995	The "utility" of the time trade-off method in cancer patients: Feasibility and proportional trade-off
J. Tsevat, E. F. Cook, M. L. Green, D. B. Matchar, N. V. Dawson, S. K. Broste, A. W. Wu, R. S. Phillips, R. K. Oye and L. Goldman	1995	Health values of the seriously ill

J. Van Der Donk, P. C. Levendag, A. J. Kuijpers, F. H. J. Roest, J. D. F. Habbema, C. A. Meeuwis and P. I. M. Schmitz	1995	Patient participation in clinical decision-making for treatment of T3 laryngeal cancer: A comparison of state and process utilities
J. K. Buckingham, J. Birdsall and J. G. Douglas	1996	Comparing three versions of the time tradeoff: Time for a change?
B. J. Cohen	1996	Assigning values to intermediate health states for cost-utility analysis: Theory and practice
P. Dolan	1996	Modelling valuations for health states: The effect of duration
P. Dolan, C. Gudex, P. Kind and A. Williams	1996	Valuing health states: A comparison of methods
M. Johannesson, B. Jonsson and G. Karlsson	1996	Outcome measurement in economic evaluation
P. F. M. Stalmeier, T. G. G. Bezembinder and I. J. Unic	1996	Proportional heuristics in time tradeoff and conjoint measurement
A. M. Stiggelbout, M. J. C. Eijkemans, G. M. Kiebert, J. Kievit, J. W. H. Leer and H. J. C. J. M. De Haes	1996	The 'utility' of the visual analog scale in medical decision making and technology assessment: Is it an alternative to the time trade-off?
G. W. Torrance, D. H. Feeny, W. J. Furlong, R. D. Barr, Y. M. Zhang and Q. N. Wang	1996	Multiattribute utility function for a comprehensive health status classification system - Health Utilities Index Mark 2
P. A. Ubel, G. Loewenstein, D. Scanlon and M. Kamlet	1996	Individual utilities are inconsistent with rationing choices: A partial explanation of why oregon's cost-effectiveness list failed
H. Bleichrodt and M. Johannesson	1997	Standard gamble, time trade-off and rating scale: Experimental results on the ranking properties of QALYs
A. E. Clarke, M. K. Goldstein, D. Michelson, A. M. Garber and L. A. Lenert	1997	The effect of assessment method and respondent population on utilities elicited for Gaucher disease
P. Dolan	1997	Modeling Valuations for EuroQol Health States
P. Dolan and M. Jones-Lee	1997	The time trade-off: A note on the effect of lifetime reallocation of consumption and discounting
P. Dolan and M. Sutton	1997	Mapping visual analogue scale health state valuations onto standard gamble and time trade-off values
R. M. Handler, L. M. Hynes and R. F. Nease Jr	1997	Effect of locus of control and consideration of future consequences on time tradeoff utilities for current health

P. F. M. Krabbe, M. L. Essink-Bot and G. J. Bonsel	1997	The comparability and reliability of five health-state valuation methods
G. C. Morrison	1997	HYE and TTO: What is the difference?
A. Robinson, P. Dolan and A. Williams	1997	Valuing health status using VAS and TTO: What lies behind the numbers?
K. Blumenschein and M. Johannesson	1998	An experimental test of question framing in health state utility assessment
K. Blumenschein and M. Johannesson	1998	Relationship between quality of life instruments, health state utilities, and willingness to pay in patients with asthma
G. B. Chapman, A. S. Elstein, T. M. Kuzel, R. Sharifi, R. B. Nadler, A. Andrews and C. L. Bennett	1998	Prostate cancer patients' utilities for health states: How it looks depends on where you stand
S. J. T. Jansen, A. M. Stiggelbout, P. P. Wakker, T. P. M. Vlieland, J. W. H. Leer, M. A. Nooy and J. Kievit	1998	Patients' utilities for cancer treatments: A study of the chained procedure for the standard gamble and time tradeoff
K. Johnston, J. Brown, K. Gerard, M. O'Hanlon and A. Morton	1998	Valuing temporary and chronic health states associated with breast screening
P. F. M. Krabbe and G. J. Bonsel	1998	Sequence effects, health profiles, and the QALY model: In search of realistic modeling
L. A. Lenert, D. J. Cher, M. K. Goldstein, M. R. Bergen and A. Garber	1998	The effect of search procedures on utility elicitations
W. Ried	1998	QALYs versus HYEs - What's fight and what's wrong. A review of the controversy
K. Stavem	1998	Quality of life in epilepsy: Comparison of four preference measures
I. Unic, P. F. M. Stalmeier, L. C. G. Verhoef and W. A. J. Van Daal	1998	Assessment of the time-tradeoff values for prophylactic mastectomy of women with a suspected genetic predisposition to breast cancer
X. Badia Llach, S. Monserrat, M. Roset and M. Herdman	1999	Feasibility, validity and test-retest reliability of scaling methods for health states: The visual analogue scale and the time trade-off

X. Badia, M. Roset and M. Herdman	1999	Inconsistent responses in three preference-elicitation methods for health states
G. B. Chapman, A. S. Elstein, T. M. Kuzel, R. B. Nadler, R. Sharifi, C. L. Bennett and G. Chapman	1999	A multi-attribute model of prostate cancer patients' preferences for health states
H. E. Fyffe, C. Deery, Z. Nugent, N. M. Nuttall and N. B. Pitts	1999	The reliability of two methods of utility assessment in dentistry
R. B. Giesler, C. M. Ashton, B. Brody, M. M. Byrne, K. Cook, J. M. Geraci, M. Hanita, J. Souchek and N. P. Wray	1999	Assessing the performance of utility techniques in the absence of a gold standard
P. A. Groome, T. A. Hutchinson, P. Tousignant and J. A. Hanley	1999	The repeatability of three methods for measuring prospective patients' values in the context of treatment choice for end-stage renal disease
L. D. Mackeigan, B. J. O'Brien and P. I. Oh	1999	Holistic versus composite preferences for lifetime treatment sequences for type 2 diabetes
C. A. McHorney	1999	Health status assessment methods for adults: Past accomplishments and future challenges
S. A. McLachlan, M. Pintilie and I. F. Tannock	1999	Third line chemotherapy in patients with metastatic breast cancer: An evaluation of quality of life and cost
C. A. E. Nickerson	1999	Assessing convergent validity of health-state utilities obtained using different scaling methods
P. F. M. Stalmeier and T. G. G. Bezembinder	1999	The discrepancy between risky and riskless utilities: A matter of framing?

Period 2

Author	Year	Title
P. Dolan	2000	Effect of age on health state valuations
C. Green, J. Brazier and M. Deverill	2000	Valuing health-related quality of life - A review of health state valuation techniques
M. Hanita	2000	Self-report measures of patient utility: should we trust them?
S. J. T. Jansen, A. M. Stiggelbout, M. A. Nooij and J. Kievit	2000	The effect of individually assessed preference weights on the relationship between holistic utilities and nonpreference-based assessment
S. J. T. Jansen, A. M. Stiggelbout, P. P. Wakker, M.	2000	Unstable preferences: A shift in valuation or an effect of the elicitation procedure?

A. Nooij, E. M. Noordijk and J. Kievit		
J. Kirsch and A. McGuire	2000	Establishing health state valuations for disease specific states: An example from heart disease
L. Lenert and R. M. Kaplan	2000	Validity and interpretation of preference-based measures of health-related quality of life
P. J. Neumann, S. J. Goldie and M. C. Weinstein	2000	Preference-based measures in economic evaluation in health care
D. Postulart and E. M. M. Adang	2000	Response shift and adaptation in chronically Ill patients
J. Souchek, J. R. Stacks, B. Brody, C. M. Ashton, R. B. Giesler, M. M. Byrne, K. Cook, J. M. Geraci and N. P. Wray	2000	A trial for comparing methods for eliciting treatment preferences from men with advanced prostate cancer: Results from the initial visit
J. S. Swan, D. G. Fryback, W. F. Lawrence, F. Sainfort, M. E. Hagenauer and D. M. Heisey	2000	A time-tradeoff method for cost-effectiveness models applied to radiology
J. Tsevat	2000	What do utilities measure?
K. F. Cook, C. M. Ashton, M. M. Byrne, B. Brody, J. Geraci, R. B. Giesler, M. Hanita, J. Souchek and N. P. Wray	2001	A psychometric analysis of the measurement level of the rating scale, time trade-off, and standard gamble
S. J. T. Jansen, J. Kievit, M. A. Nooij and A. M. Stiggelbout	2001	Stability of patients' preferences for chemotherapy: The impact of experience
M. W. Kattan, P. A. Fearn and B. J. Miles	2001	Time trade-off utility modified to accommodate degenerative and life-threatening conditions
L. A. Merlino, I. Bagchi, T. N. Taylor, P. Utrie, E. Chrischilles, W. Sumner, II, A. Mudano and K. G. Saag	2001	Preferences for fractures and other glucocorticoid-associated adverse effects among rheumatoid arthritis patients
S. Woloshin, L. M. Schwartz, M. Moncur, S. Gabriel and A. N. A. Tosteson	2001	Assessing values for health: Numeracy matters
H. Bleichrodt	2002	A new explanation for the difference between time trade-off utilities and standard gamble utilities
G. A. De Wit, M. P. Merkus, R. T. Krediet and F. T. De Charro	2002	Health profiles and health preferences of dialysis patients
P. Dolan and J. Roberts	2002	Modelling valuations for Eq-5d health states: An alternative model using differences in valuations
P. Dolan and J. Roberts	2002	To what extent can we explain time trade-off values from other information about respondents?
M. R. Gold, D. Stevenson and D. G. Fryback	2002	HALYs and QALYs and DALYs, oh my: Similarities and differences in summary measures of population health
D. Gyrd-Hansen	2002	Comparing the results of applying different methods of eliciting time preferences for health
M. Happich and B. Mazurek	2002	Priorities and prospect theory
P. F. M. Stalmeier	2002	Discrepancies between chained and classic utilities induced by anchoring with occasional adjustments
K. Stavem, I. S. Kristiansen and J. A. Olsen	2002	Association of time preference for health with age and disease severity

C. C. Sun, D. C. Bodurka, M. L. Donato, E. B. Rubenstein, C. L. Borden, K. Basen-Engquist, M. F. Munsell, J. J. Kavanagh and D. M. Gershenson	2002	Patient preferences regarding side effects of chemotherapy for ovarian cancer: Do they change over time?
H. Bleichrodt, J. L. Pinto and J. M. Abellan-Perpiñan	2003	A consistency test of the time trade-off
H. J. Schunemann, L. Griffith, D. Stubbing, R. Goldstein and G. H. Guyatt	2003	A clinical trial to evaluate the measurement properties of 2 direct preference instruments administered with and without hypothetical marker states
M. Shumway, T. Sentell, T. Chouljian, J. Tellier, F. Rozewics and M. Okun	2003	Assessing preferences for schizophrenia outcomes: Comprehension and decision strategies in three assessment methods
A. Spencer	2003	The TTO method and procedural invariance
E. A. Stolk and J. J. V. Busschbach	2003	Validity and feasibility of the use of condition-specific outcome measures in economic evaluation
L. Sung, M. L. Greenberg, N. L. Young, M. McLimont, S. Ingber, J. Rubenstein, J. Wong, T. Samanta, J. J. Doyle, A. M. Stain and B. M. Feldman	2003	Validity of a modified standard gamble elicited from parents of a hospital-based cohort of children
D. G. Dobrez and E. A. Calhoun	2004	Testing subject comprehension of utility questionnaires
J. T. King Jr, J. Tsevat and M. S. Roberts	2004	Positive association between current health and health values for hypothetical disease states
M. Locadia, P. F. M. Stalmeier, F. J. Oort, M. H. Prins, M. A. G. Sprangers and P. M. M. Bossuyt	2004	A comparison of 3 valuation methods for temporary health states in patients treated with oral anticoagulants
M. Schwarzinger, J. L. Lanoë, E. Nord and I. Durand-Zaleski	2004	Lack of multiplicative transitivity in person trade-off responses
L. Sung, N. L. Young, M. L. Greenberg, M. McLimont, T. Samanta, J. Wong, J. Rubenstein, S. Ingber, J. J. Doyle and B. M. Feldman	2004	Health-related quality of life (HRQL) scores reported from parents and their children with chronic illness differed depending on utility elicitation method
Á. Szende, K. Svensson, E. Ståhl, Á. Mészáros and G. Y. Berta	2004	Psychometric and utility-based measures of health status of asthmatic patients with different disease control level
S. M. C. Van Osch, P. P. Wakker, W. B. Van Den Hout and A. M. Stiggelbout	2004	Correcting biases in standard gamble and time tradeoff utilities
S. M. C. van Osch, P. R. Wakker, W. B. van den Hout and A. M. Stiggelbout	2004	Correcting biases in standard gamble and time tradeoff utilities
A. Bagust and S. Beale	2005	Modelling EuroQol health-related utility values for diabetic complications from CODE-2 data
L. J. Damschroder, T. R. Roberts, C. C. Goldstein, M. E. miklosovic and P. A. Ubel	2005	Trading people versus trading time: What is the difference?

J. T. King Jr, J. J. Moossy, J. Tsevat and M. S. Roberts	2005	Multimodal assessment after surgery for cervical spondylotic myelopathy
H. H. König, S. Bernert and M. C. Angermeyer	2005	Measuring preferences for depressive health states: A comparison of the EuroQol instrument, time-trade-off and contingent valuation
G. M. Lee, J. A. Salomon, C. W. LeBaron and T. A. Lieu	2005	Health-state valuations for pertussis: Methods for valuing short-term health states
J. W. Shaw, J. A. Johnson and S. J. Coons	2005	US valuation of the EQ-5D health states: Development and testing of the D1 valuation model
P. F. M. Stalmeier, J. J. V. Buusschbach, L. M. Lamers and P. F. M. Krabbe	2005	The gap effect: Discontinuities of preferences around dead
M. Van Der Pol and L. Roux	2005	Time preference bias in time trade-off
C. A. Brauer, A. B. Rosen, D. Greenberg and P. J. Neumann	2006	Trends in the measurement of health utilities in published cost-utility analyses
K. Buckingham and N. J. Devlin	2006	A theoretical framework for TTO valuations of health
B. M. Craig and S. Ramachandran	2006	Relative risk of a shuffled deck: A generalizable logical consistency criterion for sample selection in health state valuation studies
P. F. M. Krabbe	2006	Valuation structures of health states revealed with singular value decomposition
L. M. Lamers, P. F. M. Stalmeier, P. F. M. Krabbe and J. J. V. Busschbach	2006	Inconsistencies in TTO and VAS values for EQ-5D health states
R. K. Majzoub, M. Cunningham, F. Grossi, C. Maldonado, J. C. Banis and J. H. Barker	2006	Investigation of risk acceptance in hand transplantation
C. C. Reynolds, S. A. Martinez, A. Furr, M. Cunningham, J. M. Bumpous, E. J. Lentsch, J. C. Banis, D. Vasilic, B. Storey, O. Wiggins, C. Maldonado, G. Perez-Abadia and J. H. Barker	2006	Risk acceptance in laryngeal transplantation
A. Robinson and A. Spencer	2006	Exploring challenges to TTO utilities: Valuing states worse than dead
K. Schultz Hansen and L. P. Østerdal	2006	Models of quality-adjusted life years when health varies over time: Survey and analysis
D. M. Smith, R. L. Sherriff, L. Damschroder, G. Loewenstein and P. A. Ubel	2006	Misremembering colostomies? Former patients give lower utility ratings than do current patients
T. Thierer and B. Friedman	2006	Preferences for oral health states in a US community-dwelling functionally impaired older adult population: 2000-2001
A. Tsuchiya, J. Brazier and J. Roberts	2006	Comparison of valuation methods used to generate the EQ-5D and the SF-6D value sets
S. Aballéa and A. Tsuchiya	2007	Feasibility study towards valuing visual impairment using simulation spectacles
M. L. Essink-Bot, M. C. Stuifbergen, W. J. Meerding, C. W. N. Looman, G. J. Bonsel, J. J. Barendregt, G. J. Bonsel, L. G. A. Bonneux, W. B. F.	2007	Individual differences in the use of the response scale determine valuations of hypothetical health states: An empirical study

Brouwer, J. J. Van Busschbach, M. L. Essink-Bot, M. Hilhorst, P. J. Van Der Maas, W. J. Meerding and M. C. Stuifbergen		
R. M. Kaplan	2007	The future of outcomes measurement in rheumatology
L. M. Lamers	2007	The transformation of utilities for health states worse than death: Consequences for the estimation of EQ-5D value sets
P. A. McFarlane, A. Pierratos, A. M. Bayoumi and D. A. Redelmeier	2007	Estimating preference scores in conventional and home nocturnal hemodialysis patients
P. McNamee	2007	What difference does it make? The calculation of QALY gains from health profiles using patient and general population values
M. F. Janssen, E. Birnie and G. Bonsel	2008	Feasibility and reliability of the annual profile method for deriving QALYs for short-term health conditions
A. Oliver	2008	Assessing the influence of Gestalt-type characteristics on preferences over lifetime health profiles
M. Airoldi and A. Morton	2009	ADJUSTING LIFE FOR QUALITY OR DISABILITY: STYLISTIC DIFFERENCE OR SUBSTANTIAL DISPUTE?

Period 3

Author	Year	Title
A. E. Attema, H. Bleichrodt, K. I. M. Rohde and P. P. Wakker	2010	Time-tradeoff sequences for analyzing discounting and time inconsistency
A. E. Attema and W. B. F. Brouwer	2010	On the (not so) constant proportional trade-off in TTO
A. E. Attema and W. B. F. Brouwer	2010	The value of correcting values: Influence and importance of correcting TTO scores for time preference
B. M. Craig and M. Oppe	2010	From a different angle: A novel approach to health valuation
S. A. Kharroubi, A. O'Hagan and J. E. Brazier	2010	A comparison of United States and United Kingdom EQ-5D health states valuations using a nonparametric Bayesian method
R. Norman, M. T. King, D. Clarke, R. Viney, P. Cronin and D. Street	2010	Does mode of administration matter? Comparison of online and face-to-face administration of a time trade-off task
Y. Peeters, A. V. Ranchor, T. P. M. Vliet Vlieland and A. M. Stiggelbout	2010	Effect of adaptive abilities on utilities, direct or mediated by mental health?
J. W. Shaw, A. S. Pickard, S. Yu, S. Chen, V. G. Iannacchione, J. A. Johnson and S. J. Coons	2010	A median model for predicting United States population-based EQ-5D health state preferences
C. Tilling, N. Devlin, A. Tsuchiya and K. Buckingham	2010	Protocols for Time Tradeoff Valuations of Health States Worse than Dead: A Literature Review
J. Brazier, D. Rowen, A. Tsuchiya, Y. Yang and T. A. Young	2011	The impact of adding an extra dimension to a preference-based measure
L. H. Chuang and P. Kind	2011	The effect of health state selection on the valuation of EQ-5D
B. M. Craig and J. J. V. Busschbach	2011	Revisiting United States valuation of EQ-5D states

B. M. Craig and J. J. V. Busschbach	2011	Toward a more universal approach in health valuation
W. Dale, S. P. Bilir, J. Hemmerich, A. Basu, A. Elstein and D. Meltzer	2011	The prevalence, correlates, and impact of logically inconsistent preferences in utility assessments for joint health states in prostate cancer
N. J. Devlin, A. Tsuchiya, K. Buckingham and C. Tilling	2011	A uniform time trade off method for states better and worse than dead: Feasibility study of the 'lead time' approach
L. N. Ferreira, P. L. Ferreira, D. Rowen and J. E. Brazier	2011	Do Portuguese and UK health state values differ across valuation methods?
M. Garau, K. K. Shah, A. R. Mason, Q. Wang, A. Towse and M. F. Drummond	2011	Using QALYs in Cancer A Review of the Methodological Limitations
A. E. Attema and W. B. F. Brouwer	2012	The way that you do it? An elaborate test of procedural invariance of TTO, using a choice-based design
A. E. Attema and W. B. F. Brouwer	2012	Constantly Proving The Opposite? A test of CPTO using a broad time horizon and correcting for discounting
L. A. Augestad, K. Rand-Hendriksen, I. S. Kristiansen and K. Stavem	2012	Impact of transformation of negative values and regression models on differences between the UK and US EQ-5D time trade-off value sets
L. A. Augestad, K. Rand-Hendriksen, I. S. Kristiansen and K. Stavem	2012	Learning effects in time trade-off based valuation of EQ-5D health states
N. Bansback, J. Brazier, A. Tsuchiya and A. Anis	2012	Using a discrete choice experiment to estimate health state utility values
N. Bansback, A. Tsuchiya, J. Brazier and A. Anis	2012	Canadian valuation of EQ-5D health states: Preliminary value set and considerations for future valuation studies
J. Brazier, D. Rowen, Y. Yang and A. Tsuchiya	2012	Comparison of health state utility values derived using time trade-off, rank and discrete choice data anchored on the full health-dead scale
S. M. E. Finnell, A. E. Carroll and S. M. Downs	2012	The utility assessment method order influences measurement of parents' risk attitude
D. Gyrd-Hansen, T. Kjæra and J. S. Nielsen	2012	Scope insensitivity in contingent valuation studies of health care services: Should we ask twice?
K. C. Lichtendahl Jr and S. E. Bodily	2012	Multiplicative utilities for health and consumption
M. R. Lin, W. Y. Yu and S. C. Wang	2012	Examination of assumptions in using time tradeoff and standard gamble utilities in individuals with spinal cord injury
K. Rand-Hendriksen and L. A. Augestad	2012	Time trade-off and ranking exercises are sensitive to different dimensions of EQ-5D health states
K. Rand-Hendriksen, L. A. Augestad, F. A. Dahl, I. S. Kristiansen and K. Stavem	2012	A shortcut to mean-based time tradeoff tariffs for the EQ-5D?
A. E. Attema, Y. Edelaar-Peeters, M. M. Versteegh and E. A. Stolk	2013	Time trade-off: One methodology, different methods
A. E. Attema and M. M. Versteegh	2013	Would you rather be ill now, or later?
A. E. Attema, M. M. Versteegh, M. Oppe, W. B. F. Brouwer and E. A. Stolk	2013	Lead time TTO: Leading to better health state valuations?

L. A. Augestad, K. Rand-Hendriksen, K. Stavem and I. S. Kristiansen	2013	Time trade-off and attitudes toward euthanasia: Implications of using 'death' as an anchor in health state valuation
F. Augustovski, L. Rey-Ares, V. Irazola, M. Oppe and N. J. Devlin	2013	Lead versus lag-time trade-off variants: Does it make any difference?
A. Bagust	2013	Improving valuation sampling of EQ-5D health states
N. Luo, M. Li, E. A. Stolk and N. J. Devlin	2013	The effects of lead time and visual aids in TTO valuation: A study of the EQ-VT framework
L. A. Prosser, K. Payne, D. Rusinak, P. Shi and M. Messonnier	2013	Using a discrete choice experiment to elicit time trade-off and willingness-to-pay amounts for influenza health-related quality of life at different ages
K. K. Shah, A. Lloyd, M. Oppe and N. J. Devlin	2013	One-to-one versus group setting for conducting computer-assisted TTO studies: Findings from pilot studies in England and the Netherlands
P. F. M. Stalmeier and A. L. Verheijen	2013	Maximal endurable time states and the standard gamble: More preference reversals
L. Ternent and A. Tsuchiya	2013	A note on the expected biases in conventional iterative health state valuation protocols
M. M. Versteegh, A. E. Attema, M. Oppe, N. J. Devlin and E. A. Stolk	2013	Time to tweak the TTO: Results from a comparison of alternative specifications of the TTO
A. E. Attema and W. B. F. Brouwer	2014	Deriving time discounting correction factors for tto tariffs
Y. Edelaar-Peeters, A. M. Stiggelbout and W. B. Van Den Hout	2014	Qualitative and quantitative analysis of interviewer help answering the time tradeoff
Y. Gu, R. Norman and R. Viney	2014	Estimating health state utility values from discrete choice experiments - A QALY space model approach
A. B. Hauber, J. Arellano, Y. Qian, J. M. González, J. D. Posner, A. F. Mohamed, F. Gatta, B. Tombal and J. J. Body	2014	Patient preferences for treatments to delay bone metastases
S. A. Kharroubi, J. E. Brazier and Y. Yang	2014	Modeling a preference-based index for two condition-specific measures (asthma and overactive bladder) using a nonparametric bayesian method
J. T. Lauridsen, J. Lønborg, J. Gundgaard and H. H. Jensen	2014	Diminishing marginal disutility of hypoglycaemic events: results from a time trade-off survey in five countries
N. Luo, P. Wang, J. Thumboo, Y. W. Lim and H. J. M. Vrijhoef	2014	Valuation of EQ-5D-3L health states in Singapore: Modeling of time trade-off values for 80 empirically observed health states
B. Mulhern, N. Bansback, J. Brazier, K. Buckingham, J. Cairns, N. Devlin, P. Dolan, A. R. Hole, G. Kavetsos, L. Longworth, D. Rowen and A. Tsuchiya	2014	Preparatory study for the revaluation of the EQ-5D tariff: Methodology report
A. Oliver and J. Wolff	2014	Are people consistent when trading time for health?
M. Oppe, N. J. Devlin, B. Van Hout, P. F. M. Krabbe and F. De Charro	2014	A program of methodological research to arrive at the new international eq-5d-5l valuation protocol

J. Richardson, A. Iezzi, K. Sinha, M. A. Khan and J. McKie	2014	An instrument for measuring the social willingness to pay for health state improvement
F. E. Van Nooten, X. Koolman, J. J. V. Busschbach and W. B. F. Brouwer	2014	Thirty down, only ten to go?! Awareness and influence of a 10-year time frame in TTO
R. Viney, R. Norman, J. Brazier, P. Cronin, M. T. King, J. Ratcliffe and D. Street	2014	An Australian discrete choice experiment to value EQ-5D health states
L. Warshawsky-Livne, L. Novack, A. B. Rosen, S. M. Downs, J. Shkolnik-Inbar and J. S. Pliskin	2014	Gender differences in risk attitudes
B. M. Craig, S. K. Runge, K. Rand-Hendriksen, J. M. Ramos-Goñi and M. Oppe	2015	Learning and satisficing: An analysis of sequence effects in health valuation
J. L. Pinto-Prades and E. Rodríguez-Míguez	2015	The lead time tradeoff: The case of health states better than dead
B. P. Reddy, R. Adams, C. Walsh, M. Barry and P. Kind	2015	Using the Analytic Hierarchy Process to Derive Health State Utilities from Ordinal Preference Data
D. Rowen, J. Brazier and B. Van Hout	2015	A comparison of methods for converting DCE values onto the full health-dead QALY scale
F. Al Sayah, A. Mladenovic, K. Gaebel, F. Xie and J. A. Johnson	2016	How dead is dead? Qualitative findings from participants of combined traditional and lead-time time trade-off valuations
M. V. Andrade, K. Noronha, P. Kind, C. De Barros Reis and L. R. De Carvalho	2016	Logical inconsistencies in 3 preference elicitation methods for EQ-5D health states
Y. Ma, J. Huang, B. Zhu, Q. Sun, Y. Miao and H. Zou	2016	Cost-Utility Analyses of Cataract Surgery in Advanced Age-Related Macular Degeneration
L. S. Matza, K. S. Boye, D. H. Feeny, L. Bowman, J. A. Johnston, K. D. Stewart, K. McDaniel and J. Jordan	2016	The time horizon matters: results of an exploratory study varying the timeframe in time trade-off and standard gamble utility elicitation
B. Mulhern, K. Shah, M. F. Janssen, L. Longworth and R. Ibbotson	2016	Valuing Health Using Time Trade-Off and Discrete Choice Experiment Methods: Does Dimension Order Impact on Health State Values?
R. Norman, B. Mulhern and R. Viney	2016	The Impact of Different DCE-Based Approaches When Anchoring Utility Scores
D. Parkin, N. Devlin and Y. Feng	2016	What Determines the Shape of an EQ-5D Index Distribution?
J. M. Ramos-Goñi, K. Rand-Hendriksen and J. L. Pinto-Prades	2016	Does the Introduction of the Ranking Task in Valuation Studies Improve Data Quality and Reduce Inconsistencies? The Case of the EQ-5D-5L
K. Shah, B. Mulhern, L. Longworth and M. F. Janssen	2016	An empirical study of two alternative comparators for use in time trade-off studies
C. Tilling, M. Krol, A. E. Attema, A. Tsuchiya, J. Brazier, J. van Exel and W. Brouwer	2016	Exploring a new method for deriving the monetary value of a QALY

Period 4

Author	Year	Title
B. M. Craig, S. K. Runge, K. Rand-Hendriksen, J. M. Ramos-Goñi and M. Oppe	2015	Learning and satisficing: An analysis of sequence effects in health valuation
J. L. Pinto-Prades and E. Rodríguez-Míguez	2015	The lead time tradeoff: The case of health states better than dead
B. P. Reddy, R. Adams, C. Walsh, M. Barry and P. Kind	2015	Using the Analytic Hierarchy Process to Derive Health State Utilities from Ordinal Preference Data
D. Rowen, J. Brazier and B. Van Hout	2015	A comparison of methods for converting DCE values onto the full health-dead QALY scale
F. Al Sayah, A. Mladenovic, K. Gaebel, F. Xie and J. A. Johnson	2016	How dead is dead? Qualitative findings from participants of combined traditional and lead-time time trade-off valuations
M. V. Andrade, K. Noronha, P. Kind, C. De Barros Reis and L. R. De Carvalho	2016	Logical inconsistencies in 3 preference elicitation methods for EQ-5D health states
Y. Ma, J. Huang, B. Zhu, Q. Sun, Y. Miao and H. Zou	2016	Cost-Utility Analyses of Cataract Surgery in Advanced Age-Related Macular Degeneration
L. S. Matza, K. S. Boye, D. H. Feeny, L. Bowman, J. A. Johnston, K. D. Stewart, K. McDaniel and J. Jordan	2016	The time horizon matters: results of an exploratory study varying the timeframe in time trade-off and standard gamble utility elicitation
B. Mulhern, K. Shah, M. F. Janssen, L. Longworth and R. Ibbotson	2016	Valuing Health Using Time Trade-Off and Discrete Choice Experiment Methods: Does Dimension Order Impact on Health State Values?
R. Norman, B. Mulhern and R. Viney	2016	The Impact of Different DCE-Based Approaches When Anchoring Utility Scores
D. Parkin, N. Devlin and Y. Feng	2016	What Determines the Shape of an EQ-5D Index Distribution?
J. M. Ramos-Goñi, K. Rand-Hendriksen and J. L. Pinto-Prades	2016	Does the Introduction of the Ranking Task in Valuation Studies Improve Data Quality and Reduce Inconsistencies? The Case of the EQ-5D-5L
K. Shah, B. Mulhern, L. Longworth and M. F. Janssen	2016	An empirical study of two alternative comparators for use in time trade-off studies
C. Tilling, M. Krol, A. E. Attema, A. Tsuchiya, J. Brazier, J. van Exel and W. Brouwer	2016	Exploring a new method for deriving the monetary value of a QALY
F. Xie, E. Pullenayegum, K. Gaebel, N. Bansback, S. Bryan, A. Ohinmaa, L. Poissant and J. A. Johnson	2016	How different are composite and traditional TTO valuations of severe EQ-5D-5L states?
F. Al Sayah, J. A. Johnson, A. Ohinmaa, F. Xie, N. Bansback and E. Q. D. L. V. S. G. On behalf of the Canadian	2017	Health literacy and logical inconsistencies in valuations of hypothetical health states: results from the Canadian EQ-5D-5L valuation study
M. García-Molina and L. A. Chicaiza-Becerra	2017	Anchoring bias in face-to-face time-trade-off valuations of health states
K. Ludwig, J. M. G. von der Schulenburg and W. Greiner	2017	Valuation of the EQ-5D-5L with composite time trade-off for the German population - an exploratory study

N. Luo, G. Liu, M. Li, H. Guan, X. Jin and K. Rand-Hendriksen	2017	Estimating an EQ-5D-5L Value Set for China
E. L. Parsons, R. J. Stratton, A. L. Cawood, T. R. Smith and M. Elia	2017	Oral nutritional supplements in a randomised trial are more effective than dietary advice at improving quality of life in malnourished care home residents
J. M. Ramos-Goñi, M. Oppe, B. Slaap, J. J. V. Busschbach and E. Stolk	2017	Quality Control Process for EQ-5D-5L Valuation Studies
A. Robinson, A. E. Spencer, J. L. Pinto-Prades and J. A. Covey	2017	Exploring Differences between TTO and DCE in the Valuation of Health States
R. Tejwani, H. H. S. Wang, J. C. Lloyd, P. J. Kokorowski, C. P. Nelson and J. C. Routh	2017	Utility Estimation for Pediatric Vesicoureteral Reflux: Methodological Considerations Using an Online Survey Platform
F. E. van Nooten, K. Houghton, J. van Exel, M. van Agthoven, W. B. F. Brouwer and D. E. Stull	2017	A (Latent) Class of Their Own: Response Patterns in Trading Off Quantity and Quality of Life in Time Trade-Off Exercises
F. Xie, E. Pullenayegum, A. S. Pickard, J. M. Ramos Goñi, M. W. Jo and A. Igarashi	2017	Transforming Latent Utilities to Health Utilities: East Does Not Meet West
Z. Yang, J. Van Busschbach, R. Timman, M. F. Janssen and N. Luo	2017	Logical inconsistencies in time trade-off valuation of EQ-5D-5L health states: Whose fault is it?
A. Cole, K. Shah, B. Mulhern, Y. Feng and N. Devlin	2018	Valuing EQ-5D-5L health states ‘in context’ using a discrete choice experiment
B. M. Craig, K. Rand, H. Bailey and P. F. M. Stalmeier	2018	Quality-Adjusted Life-Years without Constant Proportionality
Y. Feng, N. J. Devlin, K. K. Shah, B. Mulhern and B. van Hout	2018	New methods for modelling EQ-5D-5L value sets: An application to English data
Y. Feng, A. R. Hole, M. Karimi, A. Tsuchiya and B. van Hout	2018	An exploration of the non-iterative time trade-off method to value health states
M. F. Jonker, B. Donkers, E. W. de Bekker-Grob and E. A. Stolk	2018	Advocating a Paradigm Shift in Health-State Valuations: The Estimation of Time-Preference Corrected QALY Tariffs
S. A. Kharroubi	2018	Valuations of EQ-5D health states: could United Kingdom results be used as informative priors for the United States
S. A. Kharroubi and C. A. Daher	2018	Modelling a preference-based index for EQ-5D using a non-parametric Bayesian method
S. Lim, M. F. Jonker, M. Oppe, B. Donkers and E. Stolk	2018	Severity-Stratified Discrete Choice Experiment Designs for Health State Evaluations
M. D. Oliveira, A. Agostinho, L. Ferreira, P. Nicola and C. Bana E Costa	2018	Valuing health states: Is the MACBETH approach useful for valuing EQ-5D-3L health states?
J. M. Ramos-Goñi, B. M. Craig, M. Oppe, Y. Ramallo-Fariña, J. L. Pinto-Prades, N. Luo and O. Rivero-Arias	2018	Handling Data Quality Issues to Estimate the Spanish EQ-5D-5L Value Set Using a Hybrid Interval Regression Approach
E. L. Y. Wong, J. M. Ramos-Goñi, A. W. L. Cheung, A. Y. K. Wong and O. Rivero-Arias	2018	Assessing the Use of a Feedback Module to Model EQ-5D-5L Health States Values in Hong Kong

E. L. Y. Wong, K. Shah, A. W. L. Cheung, A. Y. K. Wong, M. Visser and E. Stolk	2018	Evaluation of Split Version and Feedback Module on the Improvement of Time Trade-Off Data
J. E. Brazier, D. Rowen, A. Lloyd and M. Karimi	2019	Future Directions in Valuing Benefits for Estimating QALYs: Is Time Up for the EQ-5D?
R. M. Kaplan, C. M. Crespi, E. Dahan, J. D. Saucedo, C. Pagan and C. S. Saigal	2019	Comparison of Rating Scale, Time Tradeoff, and Conjoint Analysis Methods for Assessment of Preferences in Prostate Cancer
M. Karimi, J. Brazier and S. Paisley	2019	Effect of Reflection and Deliberation on Health State Values: A Mixed-Methods Study
S. A. Kharroubi and D. Rowen	2019	Valuation of preference-based measures: can existing preference data be used to select a smaller sample of health states?
S. A. Lipman, W. B. F. Brouwer and A. E. Attema	2019	QALYs without bias? Nonparametric correction of time trade-off and standard gamble weights based on prospect theory
S. A. Lipman, W. B. F. Brouwer and A. E. Attema	2019	The Corrective Approach: Policy Implications of Recent Developments in QALY Measurement Based on Prospect Theory
L. S. Matza, J. E. Brazier, K. D. Stewart, L. Pinto, R. H. Bender, L. Kircik, J. Jordan, K. J. Kim, A. Mutebi, H. N. Viswanathan and A. Menter	2019	Developing a preference-based utility scoring algorithm for the Psoriasis Area Severity Index (PASI)
J. L. Pinto-Prades, N. McHugh, C. Donaldson and S. Manoukian	2019	Sequence effects in time trade-off valuation of hypothetical health states
E. M. Pullenayegum, A. S. Pickard and F. Xie	2019	Latent Class Models Reveal Poor Agreement between Discrete-Choice and Time Tradeoff Preferences
J. Shen, M. Breckons, L. Vale, R. Pickard and O. t. i. for the	2019	Using Time Trade-Off Methods to Elicit Short-Term Utilities Associated with Treatments for Bulbar Urethral Stricture
J. Shen, S. Hill, D. Mott, M. Breckons, L. Vale and R. Pickard	2019	Conducting a Time Trade-Off Study Alongside a Clinical Trial: A Case Study and Recommendations
A. Spencer, E. Tomeny, R. E. Mujica-Mota, A. Robinson, J. Covey and J. L. Pinto-Prades	2019	Do time trade-off values fully capture attitudes that are relevant to health-related choices?
Z. Yang, N. Luo, M. Oppe, G. Bonsel, J. Busschbach and E. Stolk	2019	Toward a Smaller Design for EQ-5D-5L Valuation Studies
A. E. Attema, H. Bleichrodt, O. l'Haridon and S. A. Lipman	2020	A comparison of individual and collective decision making for standard gamble and time trade-off
L. A. Augestad, K. Rand, N. Luo and M. Barra	2020	Using the Choice Sequence in Time Trade-Off as Discrete Choices: Do the Two Stories Match?
S. Hao, E. Heintz, G. Helgesson, S. Langenskiöld, J. Chen and K. Burström	2020	Influence of elicitation procedure and phrasing on health state valuations in experience-based time trade-off tasks among diabetes patients in China
M. Hernandez Alava, S. Pudney and A. Wailoo	2020	The EQ-5D-5L Value Set for England: Findings of a Quality Assurance Program
M. Jakubczyk and D. Golicki	2020	Elicitation and modelling of imprecise utility of health states

S. A. Lipman, W. B. F. Brouwer and A. E. Attema	2020	Living up to expectations: Experimental tests of subjective life expectancy as reference point in time trade-off and standard gamble
L. Panattoni, C. E. Phelps, T. A. Lieu, S. Alexeeff, S. O'Neill, J. S. Mandelblatt and S. D. Ramsey	2020	Feasibility of Measuring Preferences for Chemotherapy Among Early-Stage Breast Cancer Survivors Using a Direct Rank Ordering Multicriteria Decision Analysis Versus a Time Trade-Off
B. Roudijk, A. R. T. Donders and P. F. M. Stalmeier	2020	A Head-On Ordinal Comparison of the Composite Time Trade-Off and the Better-Than-Dead Method
K. K. Shah, J. M. Ramos-Goñi, S. Kreimeier and N. J. Devlin	2020	An exploration of methods for obtaining 0 = dead anchors for latent scale EQ-5D-Y values
L. Vallejo-Torres, B. García-Lorenzo, O. Rivero-Arias and J. L. Pinto-Prades	2020	The societal monetary value of a QALY associated with EQ-5D-3L health gains
X. Wang, L. Zhuo, Y. Ma, T. Cai, A. Must, L. Xu and L. Zhuo	2020	Similar responses to EQ-5D-3L by two elicitation methods: Visual analogue scale and time trade-off
I. Waudby-Smith, A. S. Pickard, F. Xie and E. M. Pullenayegum	2020	Using Both Time Tradeoff and Discrete Choice Experiments in Valuing the EQ-5D: Impact of Model Misspecification on Value Sets
M. F. Janssen, E. Birnie and G. J. Bonsel	2021	A Head-to-Head Comparison of the Standard Quality-Adjusted Life Year Model With the Annual Profile Model
R. Jiang, T. Kohlmann, T. A. Lee, A. Mühlbacher, J. Shaw, S. Walton and A. S. Pickard	2021	Increasing respondent engagement in composite time trade-off tasks by imposing three minimum trade-offs to improve data quality
J. S. Nielsen, D. Gyrd-Hansen and T. Kjær	2021	Sample restrictions and the elicitation of a constant willingness to pay per quality adjusted life year
J. Wu, S. Xie, X. He, G. Chen, G. Bai, D. Feng, M. Hu, J. Jiang, X. Wang, H. Wu, Q. Wu and J. E. Brazier	2021	Valuation of SF-6Dv2 Health States in China Using Time Trade-off and Discrete-Choice Experiment with a Duration Dimension
